# Digital Technology Interventions for Risk Factor Modification in Patients With Cardiovascular Disease: Systematic Review and Meta-analysis

**DOI:** 10.2196/21061

**Published:** 2021-03-03

**Authors:** Adewale Samuel Akinosun, Rob Polson, Yohanca Diaz - Skeete, Johannes Hendrikus De Kock, Lucia Carragher, Stephen Leslie, Mark Grindle, Trish Gorely

**Affiliations:** 1 Department of Nursing and Midwifery, Institute of Health Research and Innovation Centre for Health Science University of the Highlands and Islands Inverness United Kingdom; 2 Highland Health Sciences Library Centre for Health Science University of the Highlands and Islands Inverness United Kingdom; 3 School of Health and Science Dundalk Institute of Technology Dundalk Ireland; 4 Cardiology Unit Raigmore Hospital NHS Highlands Inverness United Kingdom

**Keywords:** digital technologies, mHealth, eHealth, risk factors, cardiovascular diseases, telehealth, cardiac rehabilitation, behavior, systematic review, meta-analysis, mobile phone

## Abstract

**Background:**

Approximately 50% of cardiovascular disease (CVD) cases are attributable to lifestyle risk factors. Despite widespread education, personal knowledge, and efficacy, many individuals fail to adequately modify these risk factors, even after a cardiovascular event. Digital technology interventions have been suggested as a viable equivalent and potential alternative to conventional cardiac rehabilitation care centers. However, little is known about the clinical effectiveness of these technologies in bringing about behavioral changes in patients with CVD at an individual level.

**Objective:**

The aim of this study is to identify and measure the effectiveness of digital technology (eg, mobile phones, the internet, software applications, wearables, etc) interventions in randomized controlled trials (RCTs) and determine which behavior change constructs are effective at achieving risk factor modification in patients with CVD.

**Methods:**

This study is a systematic review and meta-analysis of RCTs designed according to the PRISMA (Preferred Reporting Items for Systematic Reviews and Meta-analysis) statement standard. Mixed data from studies extracted from selected research databases and filtered for RCTs only were analyzed using quantitative methods. Outcome hypothesis testing was set at 95% CI and *P*=.05 for statistical significance.

**Results:**

Digital interventions were delivered using devices such as cell phones, smartphones, personal computers, and wearables coupled with technologies such as the internet, SMS, software applications, and mobile sensors. Behavioral change constructs such as cognition, follow-up, goal setting, record keeping, perceived benefit, persuasion, socialization, personalization, rewards and incentives, support, and self-management were used. The meta-analyzed effect estimates (mean difference [MD]; standard mean difference [SMD]; and risk ratio [RR]) calculated for outcomes showed benefits in total cholesterol SMD at −0.29 [−0.44, −0.15], *P*<.001; high-density lipoprotein SMD at –0.09 [–0.19, 0.00], *P*=.05; low-density lipoprotein SMD at −0.18 [−0.33, −0.04], *P*=.01; physical activity (PA) SMD at 0.23 [0.11, 0.36], *P*<.001; physical inactivity (sedentary) RR at 0.54 [0.39, 0.75], *P*<.001; and diet (food intake) RR at 0.79 [0.66, 0.94], *P*=.007. Initial effect estimates showed no significant benefit in body mass index (BMI) MD at −0.37 [−1.20, 0.46], *P*=.38; diastolic blood pressure (BP) SMD at −0.06 [−0.20, 0.08], *P*=.43; systolic BP SMD at −0.03 [−0.18, 0.13], *P*=.74; Hemoglobin A_1C_ blood sugar (HbA_1c_) RR at 1.04 [0.40, 2.70], *P*=.94; alcohol intake SMD at −0.16 [−1.43, 1.10], *P*=.80; smoking RR at 0.87 [0.67, 1.13], *P*=.30; and medication adherence RR at 1.10 [1.00, 1.22], *P*=.06.

**Conclusions:**

Digital interventions may improve healthy behavioral factors (PA, healthy diet, and medication adherence) and are even more potent when used to treat multiple behavioral outcomes (eg, medication adherence plus). However, they did not appear to reduce unhealthy behavioral factors (smoking, alcohol intake, and unhealthy diet) and clinical outcomes (BMI, triglycerides, diastolic and systolic BP, and HbA_1c_).

## Introduction

### Background

Cardiovascular diseases (CVDs), including coronary heart disease (CHD), stroke, and peripheral vascular disease, remain one of the most common causes of early death and disability worldwide, with 17.9 million deaths and 422.7 million cases each year [1]. In 2017 alone, there were approximately 1.7 million inpatient episodes of CVDs in the United Kingdom [2]. This imposes a heavy burden on individuals and the society, accounting for £19 billion in public expense, 7.4 million disabilities, and 167,000,000 deaths in 2019 [1].

Although there are genetic, demographic, and environmental causes of CVDs [3], approximately 50% of CVD risk is attributable to modifiable lifestyle factors such as obesity, diabetes, inactivity, and smoking [4]. However, despite widespread education and personal knowledge, many individuals fail to adequately modify these risk factors, even after a cardiovascular event with cardiac rehabilitation care center support [4]. Failure to address this challenge (ie, a change from cognitive insight to manifest action) results in patients remaining at a higher risk of future cardiovascular events with associated personal, social, and economic costs.

There could be several reasons for the challenges in the personalized management (ie, modification) of CVD and other chronic disease risk factors. These include care center accessibility and outpatient mobility and morbidity, comprehensibility, and retainability [5]. For these reasons, lifestyle risk factor management (particularly low physical activity [PA] and obesity) remains suboptimally addressed in CVD outpatients [6]. These show that although there is a modest success with rehabilitation care center interventions, the technical reach of this population-based approach is limited in its ability to bring about a significant sustainable change in exposed individuals [5].

To achieve a sustainable change, social construct strategies (eg, self-management, motivation, perceived benefits, etc) embedded in behavioral change interventions have shown health benefits in chronic disease risk factor management [7]. Their substantial contribution to changes in health behaviors suggests a worthy consideration in behavioral health interventions at the individual level. However, in the context of a population-based health behavior change in rehabilitation care centers, there are limitations in the ability of social constructs to make an individual cope sustainably with its strategies at the personal level [6].

The emergence of digital health technologies (eg, the internet, phone apps and devices, and wearable sensors for telemedicine, web browsing, emailing, text messaging, monitoring) in the health care sector [8], designed to manage and monitor chronic disease lifestyle factors, has shown potential in personalized chronic disease lifestyle factor modification [9]. This potential is based on evidence that healthy lifestyle factors are behavior-specific, measurable, and modifiable [10]. Due to the commercial drive and attributed qualities, many of these technologies and devices have been continually used in cardiac rehabilitation care centers, and even instead of care [11,12]. However, despite their popularity and potential, these technologies lack evidence summary of secondary prevention of clinically relevant outcomes, which result from behavior change in CVD outpatients, especially at a personalized level [12,13].

### Objectives

The primary objective of this systematic review is to identify and measure the effectiveness of digital technology (eg, mobile phones, the internet, software applications, wearables, etc) interventions in randomized controlled trials (RCTs) and determine which behavior change constructs were effective at achieving risk factor modification among patients with CVD.

## Methods

### Study Design

This study is a systematic literature review and meta-analysis of RCTs designed in line with the PRISMA (Preferred Reporting Items for Systematic Reviews and Meta-analysis) statement standard [14]. The protocol was registered with PROSPERO protocol ID CRD42019139801 [15].

### Inclusion Criteria for Considering Studies in This Review

#### Types of Studies

RCTs of digital interventions with a minimum of 4 weeks of intervention follow-up period were considered. Publications in English from 2000 to September 2019 were included.

#### Types of Participants

The considered studies focused on an adult population (≥18 years) with a minimum of 30 participants in the intervention study.

#### Types of Interventions

The considered intervention types were digital intervention only versus usual care or digital intervention plus usual care versus usual care. The studies were required to be based on a well-defined CVD risk factor modification measurement function and intention to modify health behavior in outpatients diagnosed or treated for CVD only or with comorbidities using a named digital device. 

#### Types of Outcome Measures

Clinical and behavioral outcomes were measured at baseline and endpoint. All outcome hypothesis testing was set at 95% CI and a 2-tailed *P* value of .05 level of statistical significance. Behavioral outcomes included PA (physical inactivity [PI], sedentary lifestyle), food intake (diet), smoking, alcohol intake, and medication adherence. Clinical outcomes included BMI, cholesterol levels (total cholesterol [TC], high-density lipoproteins [HDLs], low-density lipoproteins [LDLs], and triglycerides [TGs]), blood pressure (diastolic BP and systolic BP), and blood sugar levels (HbA_1c_), which are measures of obesity, hypercholesterolemia, hypertension, and diabetes, respectively.

### Exclusion Criteria

Studies related to non-RCTs, nondigital interventions, and journal papers not published in English were excluded. In addition, studies whose populations shared modifiable risk factors with CVD but were not diagnosed with a named CVD; studies with nonclinical or nonbehavioral outcomes, such as studies with genetic outcomes and studies directly measuring hospital or staff service efficiency, etc; and healthy population or intensive care studies were excluded.

No analysis was conducted on data of outcomes from subgroups (endpoint to endpoint) within the population of the included studies.

### Search Methods for Identification of Studies

A single 6S pyramid systematic literature search strategy was developed (Table S3 in the Multimedia Appendix 1). This was run on Ovid Medline and Ovid Embase, Web of Science (Core Collection), Scopus, Cochrane Library, and on the following databases in EBSCOHost: CINAHL, Psych Info, Health Source, Open Dissertation, Psych Article, and Business Source Elite. Filters were used to narrow searches to studies using RCT methodology and those written in the English language and from the year 2000 onward. The year limit was applied, in line with the World Health Organization’s release of the document on the approach to digital health strategies [16], as the start of the mass availability and use of digital technologies, making pre-2000 literature less relevant.

Two independent reviewers (AS and RP) were involved in a thorough search strategy build-up and study extraction to identify potentially relevant publications. References and citations were also searched. Where an abstract did not provide sufficient precision to meet the selection prerequisite, the article was reserved for full-text review. Relevant articles retrieved for full-text reviews were independently evaluated (AS and MG). The consensus to include or exclude a trial was reached based on study design, method, population demography, intervention mechanism, and study outcomes.

### Data Collection and Analysis

#### Data Collection

The PRISMA search protocol [14] was followed, with all extracted data subsequently managed using the Mendeley Desktop reference manager software (Elsevier). Publication search outcomes were imported in .ris format into the Mendeley Desktop and partitioned based on the search database source. Imported publications were autochecked for duplicates using the software, and a further manual, independent duplication check was carried out (AS and YD). Publication papers were title-read, abstract-read, and full-text read based on the inclusion and exclusion preselection criteria. Selected journal papers were read for data synthesis and analysis.

#### Data Extraction and Management

Data were extracted into a preset Excel (Microsoft Corporation) worksheet. The data extraction process was performed independently (AS) using predetermined variables and then validated accordingly (MG). Data extracted included population demographics (mean age, sex, size, and CVD diagnosis) description of the study (authors, year of publication, country, intervention acronym, digital device, intervention type, trial protocol registration, design, and duration), behavioral change context (change technique and risk factors), and clinical study outcomes (outcome measures, outcome units, mean baseline measurements, mean outcome measurements, *P* values, and SDs). Authors of studies with insufficient or missing outcome data were contacted for further information.

#### Data Analysis

All extracted data from the selected studies were analyzed (Table 1) using Review Manager 5.3 (The Cochrane Collaboration). An assessment of the risk of bias (Table 2) was carried out by 2 researchers, AS and YD, using the modified Cochrane Collaboration AUB KQ1 Risk of Bias Assessment Tool, Review Manager 5.3 (The Cochrane Collaboration) with assessment result validation by an external independent researcher. Bias quality was assessed as high, low, or unclear for individual elements from 6 items: selection, performance, attrition, reporting, proportion, outcome, and treatment efficacy. Where the attrition bias risk is high, there is more likely to be a high treatment efficacy bias except where the basis for participant dropout is a medical reason, relocation, or death. Quality assessment items were evaluated by an external assessor to validate the initial scales judged by the author. Controversial evaluation differences were discussed, and consensus was reached before the final documentation. The risk of bias across studies was assessed for each analyzed outcome for publication bias reporting. Results were generated with meta-analysis data for each outcome and are presented in the Results section.

The review authors considered the variations in outcome measurement across studies by applying appropriate statistical methods (fixed effect and random effect) using the Inverse-Variance and Mantel-Haenszel (DerSimonian and Laird) models to generate meta-analytic estimates of treatment effect using the Review Manager 5.3 software. Differences in effects were examined by comparing digital with usual care. The weighted mean difference (MD) or standardized mean difference (SMD) was calculated for continuous data using the inverse variance statistical method. Relative risks were calculated for dichotomous data using the Mantel-Haenszel statistical method (The Cochrane Collaboration). Provision for variations among the included studies was made using the random effect meta-analysis model in analyzing all included studies. The heterogeneity statistic I^2^ was calculated to describe the percentage of variation among the studies. Hypothesis testing was set at a 2-tailed 0.05 level of significance and a 95% CI. No analysis was conducted on the data of outcomes from subgroups within the population of the included studies.

Sensitivity analyses were proportionately conducted on outcomes to check the cumulative effects of the publication year, participant size, efficacy, and category of intervention (risk factors and digital intervention) on statistical significance. For food intake, studies with interventions targeting healthy diet and studies targeting unhealthy diet were analyzed separately to provide a clearer insight into treatment effects. Studies with treatment for medication adherence were analyzed separately for (1) other risk factor treatments plus medication adherence using the SMS text messaging intervention only, (2) medication adherence treatment only (with no other risk factor) using the SMS text messaging intervention alone, (3) other risk factor treatment plus medication adherence treatment using non-SMS text messaging intervention, and (4) medication adherence treatment with SMS text messaging intervention only. The results are presented and discussed in the Results and Discussion sections, respectively.

**Table 1:** Summary of meta-analysis results^a^.

Outcomes or subgroups	Number of studies (N=25), n (%)	Participants	Statistical methods	Effect estimates (95% CI)	*P* value
BMI	10 (40)	2558	MD^b^ (IV, random, 95% CI)	−0.37 (−1.20 to 0.46)	.38
Total cholesterol	9 (36)	1783	SMD^c^ (IV, random, 95% CI)	−0.29 (−0.44 to −0.15)	<.001
High-density lipoprotein	9 (36)	1783	SMD (IV, random, 95% CI)	−0.09 (−0.19 to 0.00)	.05
Low-density lipoprotein	12 (48)	3431	SMD (IV, random, 95% CI)	−0.18 (−0.33 to −0.04)	.01
Triglycerides	8 (32)	1660	SMD (IV, random, 95% CI)	−0.07 (−0.24 to 0.11)	.28
Diastolic BP^d^	11 (44)	2460	SMD (IV, random, 95% CI)	−0.06 (−0.20 to 0.08)	.43
Systolic BP	12 (48)	3283	SMD (IV, random, 95% CI)	−0.03 (−0.18 to 0.13)	.74
Physical activity	14 (56)	3015	SMD (IV, random, 95% CI)	0.23 (0.11 to 0.36)	<.001
Alcohol consumption	4 (16)	651	SMD (IV, random, 95% CI)	−0.16 (−1.43 to 1.10)	.80
Blood sugar, HbA_1c_^e^	2 (8)	380	RR^f^ (M-H, random, 95% CI)	1.04 (0.40 to 2.70)	.94
Physical inactivity	4 (16)	1054	RR (M-H, random, 95% CI)	0.54 (0.39 to 0.75)	<.001
Food intake (diet)	6 (24)	716	RR (M-H, random, 95% CI)	0.79 (0.66 to 0.94)	.007
Health diet	3 (12)	173	RR (M-H, random, 95% CI)	0.70 (0.55 to 0.89)	.004
Unhealthy diet	3 (12)	185	RR (M-H, random, 95% CI)	0.90 (0.68 to 1.19)	.47
Smoking	11 (44)	2916	RR (M-H, random, 95% CI)	0.87 (0.67 to 1.13)	.30
Medication adherence	11 (44)	2710	RR (M-H, random, 95% CI)	1.10 (1.00 to 1.22)	.06
Medication adherence (multiple treatment)	5 (20)	758	RR (M-H, random, 95% CI)	1.07 (1.01 to 1.14)	.02

^a^Summary of analyzed data.

^b^MD: mean difference.

^c^SMD: standard mean difference.

^d^BP: blood pressure.

^e^HbA_1c_: Hemoglobin A_1c_ blood sugar.

^f^RR: risk ratio.

**Table 2:** Risk of bias in included studies^a^.

Study (reference)	Selection bias		Performance bias	Detection bias	Attrition bias	Proportion bias	Outcome bias	Reportin bias	Treatmen efficacy
	Random sequence generation	Allocation concealment	Blinding of participants and personnel	Blinding of outcome assessment	Incomplete outcome data	Groups balanced at baseline	Groups received same intervention	Selective reporting	Intention-to-treat analysis
Akhu-Z et al, 2016 [17]	Low	Unclear	Unclear	Low	High	Low	Low	Low	High
Chow et al, 2015 [18]	Low	Low	Low	Low	High	Low	High	Low	High
Dale et al, 2015 [19]	Low	Low	Low	High	Low	High	High	Low	Low
Devi et al, 2014 [20]	Low	Low	Low	High	High	Low	Low	High	Unclear
Frederix et al, 2015 [21]	Low	Low	Low	Low	High	Low	Low	Low	Unclear
Hawkes et al, 2012 [22]	Low	High	Low	Low	High	Low	Low	High	Unclear
Johnston et al, 2016 [23]	Low	Unclear	Unclear	Unclear	High	High	High	High	Unclear
Kamal et al, 2015 [24]	Low	Low	Low	Low	High	Low	High	Low	High
Khonsari et al, 2015 [25]	Low	Unclear	High	Unclear	Low	Low	High	Low	Low
Kraal et al, 2014 [26]	Low	Low	Unclear	Unclear	Low	Low	High	Low	Low
Lear et al, 2014 [27]	Low	Low	Low	Low	High	Low	Low	Low	Low
Maddison et al, 2014 [28]	Low	Low	Low	Low	High	Low	High	Low	Unclear
Ogren et al, 2018 [29]	Low	Low	High	Unclear	Low	Low	Low	Unclear	High
Pandey et al, 2014 [30]	Unclear	Unclear	Low	Low	Unclear	Low	Low	Unclear	Unclear
Park et al, 2013 [31]	Low	Low	High	Low	Low	Unclear	Low	Low	Low
Quilici et al, 2012 [32]	Low	Low	Unclear	Unclear	High	Low	Low	Low	Unclear
Redfern et al, 2009 [33]	Low	Low	Low	Low	Low	Low	Low	Low	Low
Reid et al, 2011 [34]	Low	Low	Low	Low	High	Low	Low	Low	Low
Southard et al, 2003 [35]	Low	Low	Low	Low	Low	Low	Low	Low	Low
Tiede et al, 2017 [36]	Low	Low	High	Unclear	High	High	Low	Low	High
Vale et al, 2002 [37]	Low	High	High	Low	High	Low	Low	High	Low
Vemooij et al, 2012 [38]	Low	Low	Unclear	Unclear	Low	Low	High	Low	Low
Wan et al, 2016 [39]	Low	Low	Low	Low	High	Low	Low	Low	Low
Widmer et al, 2017 [40]	Low	Low	Low	Low	Low	High	High	Low	Unclear
Zheng et al, 2019 [41]	Low	High	Low	Low	Low	Low	High	Low	Low

^a^Risk of bias: review authors’ judgments about each risk of bias item for the included studies.

## Results

### Search Results

**Figure 1:**
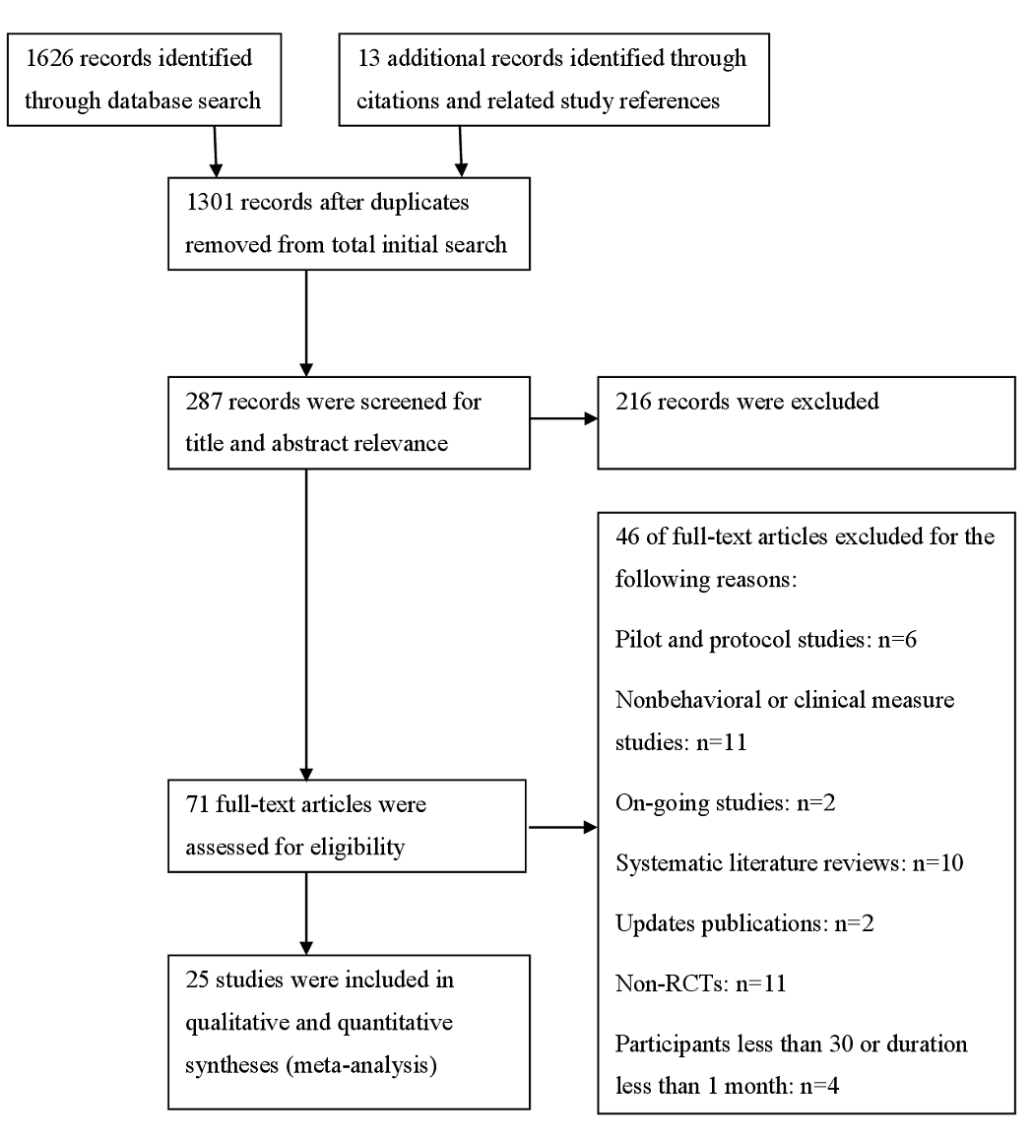
Database search flowchart. RCT: randomized controlled trial.

The search retrieved 1626 papers with the auto-removal of 326 duplicates. A total of 35 papers remained after applying inclusion and exclusion criteria. Ten papers were excluded because they were systematic literature reviews but not RCTs. A final count of 25 papers was considered for the review: 12 from the database searches and 13 from references, citations, and gray literature (Figure 1). The included studies are listed in the table of included studies (Table S4 in Multimedia Appendix 1), and excluded studies are listed in the table of excluded studies (Table S5 in Multimedia Appendix 1) with reasons.

### Study Characteristics

Studies are described by their common characteristics, which include population demography, digital technologies and brands, intervention mechanisms and behavioral change constructs, types of CVD, and general characteristics. Table S6 (Multimedia Appendix 2) provides a detailed summary of the reviewed studies.

#### Population Demography

The included studies had a total participant count of 5,779 at baseline, with a mean age of 60.03 years (SD 2.73) and a male proportion of 75.22% (4347/5779). A geographical analysis of the included studies identified evenly distributed locations of studies on a global scale, with countries spanning Europe (5 studies), Middle East (2 studies), Asia (3 studies), Northern America (6 studies), Scandinavia (2 studies), Australasia (6 studies), and the United Kingdom (1 study).

#### Digital Technologies and Brands 

Cardiovascular digital interventions were delivered using devices such as cell phones, smartphones, personal computers (laptops and desktops), and wearables. Technologies included the internet, software applications, and mobile sensors. Intervention device brand names such as Personal Health Assistant, PHA, FIT@Home, HeartLinks, SUPPORT, SMS4Stroke, ProActive Heart, Text4Heart, CHAT, CardioFit, HEART, ActivateYourHeart, MEMS, vCRP, COACH, CHOICE, and TBHC were recorded.

#### Intervention Mechanisms and Behavioral Change Constructs

Intervention mechanisms (ie, the digital strategy plus behavioral construct) were based on online support, telerehabilitation, telemonitoring, and online coaching. The interventions included major behavioral change constructs such as cognition, follow-up, goal setting, record keeping, perceived benefit, persuasion, social engagement (virtual), personalization (or customization), rewards and incentives, support, and self-management.

CVD types: Diagnosed CVDs included CHD, coronary artery disease, myocardial infarction, acute coronary syndrome, angina, atherosclerosis, heart failure, transient ischemic attack, and stroke. Four studies [17,27,35,42] were not specific about CVD diagnosis in the study population.

#### General Characteristics 

Study follow-up ranged from 1 to 4 (9 studies), 6 (12 studies), 12 (3 studies), and 24 months (1 study), with 6 months being the most frequent duration of the follow-up period for interventions. No data on outcomes from subgroups within the populations of the included studies were considered in the analysis.

The main units of outcome measurements were kg/m^2^ (BMI), mg/dL, and mmol/L (TC, HDL, low-density lipoprotein [LDL], and TGs, mmHg (diastolic blood pressure [DBP] and systolic blood pressure [SBP]), min/week (PA and PI), percentage, % (Hemoglobin A_1C_ blood sugar—HbA_1c_, alcohol, smoking, and food intake), and Morisky Medication Adherence Scale for Medication Adherence 8. In the treatment context, all the intervention studies had been administered either as digital intervention versus usual care (15 studies) or digital intervention and usual care versus usual care (10 studies). 

### Synthesis of Results

The use of digital intervention compared with usual care significantly modified all CVD risk factors except BMI, TG, DBP, SBP, HbA_1c_, alcohol intake, smoking, and medication adherence. A detailed summary of these findings is presented in Table 1.

### Summary of Results

Effect estimates (MD, SMD, and RR) were significant and in favor of digital interventions for TC, HDL, LDL, PA, PI, and food intake.

#### Clinical Outcomes

**Figure 2:**
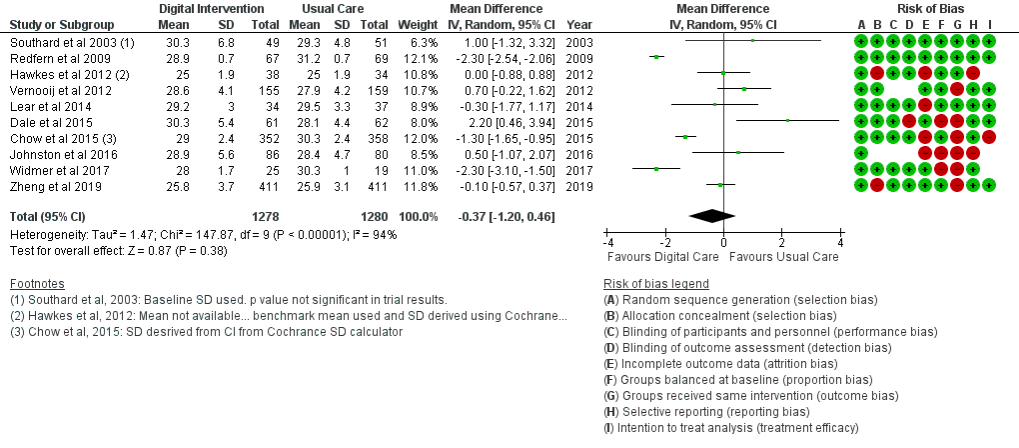
Outcomes of the examined studies for BMI.

**Figure 3:**
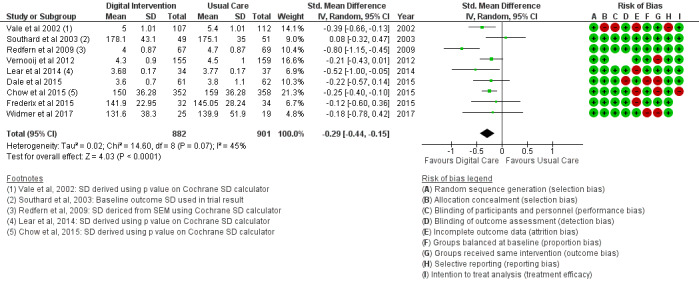
Outcomes of the examined studies for total cholesterol.

**Figure 4:**
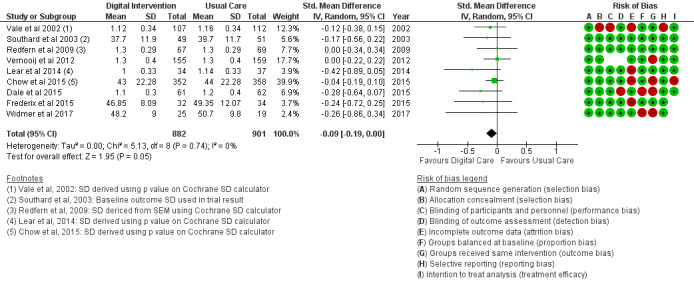
Outcomes of the examined studies for high-density lipoprotein.

**Figure 5:**
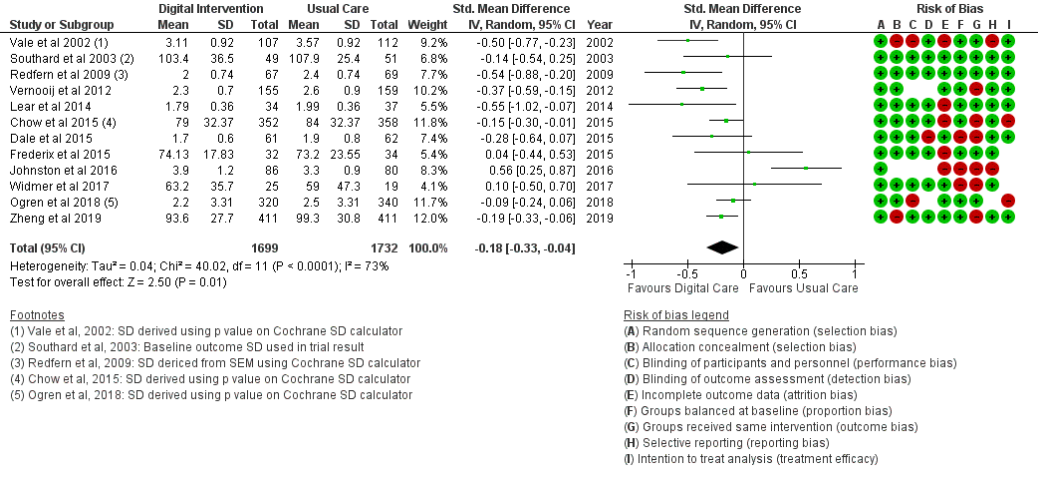
Outcomes of the examined studies for low-density lipoprotein.

**Figure 6:**
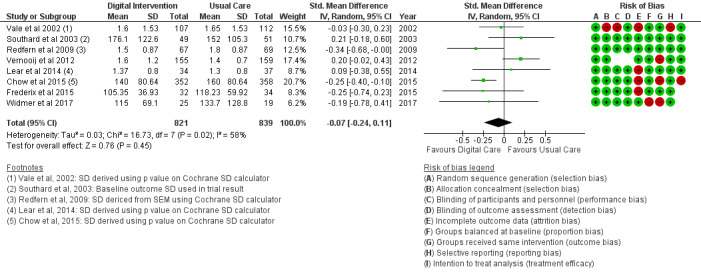
Outcomes of the examined studies for triglycerides.

**Figure 7:**
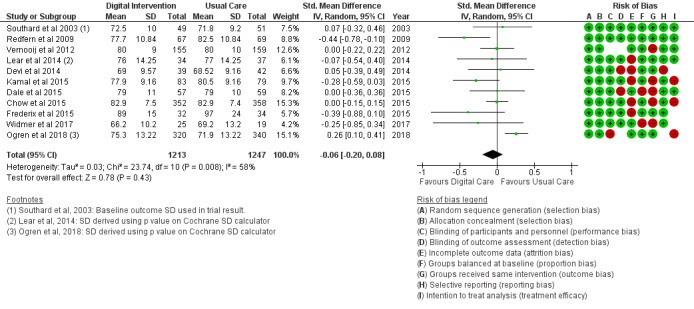
Outcomes of the examined studies for diastolic blood pressure.

**Figure 8:**
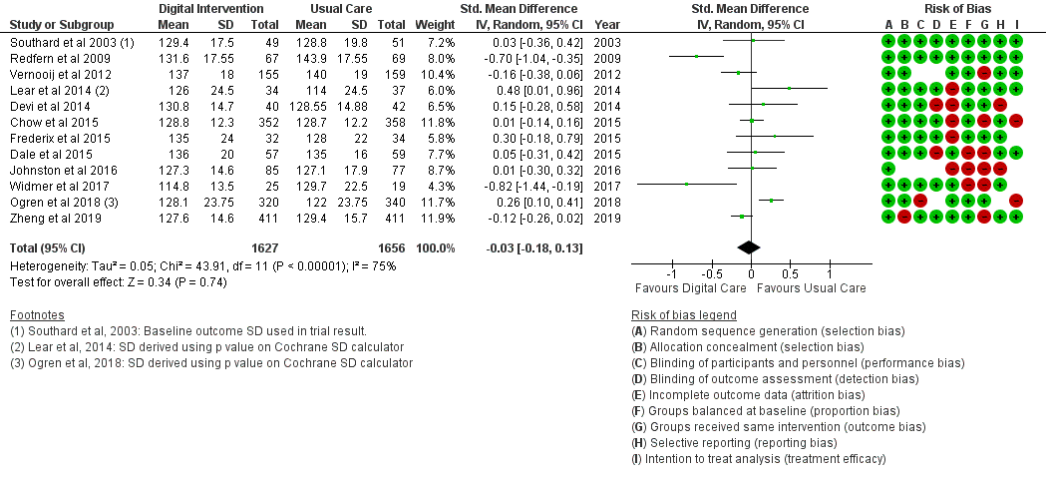
Outcomes of the examined studies for systolic blood pressure.

**Figure 9:**
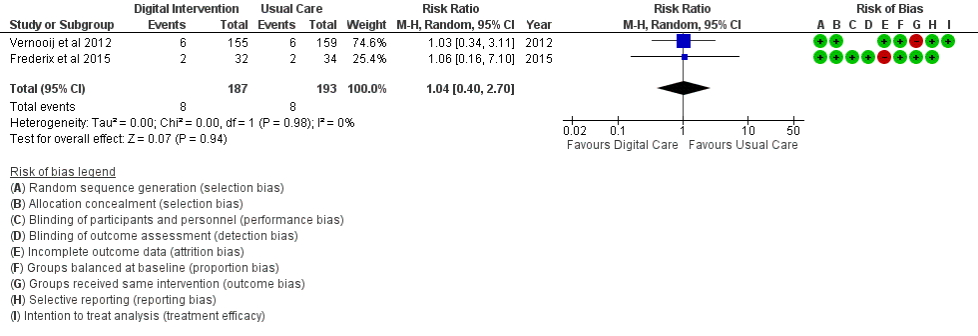
Outcomes of the examined studies for blood sugar HbA_1c_.

The BMI outcome (Figure 2) reported MD was estimated at −0.37 (95% CI –1.20 to 0.46, *P*=.38). The TC outcome (Figure 3) reported an SMD estimated at −0.29 (95% CI –0.44 to –0.15, *P*<.001). The HDL outcome (Figure 4) reported an SMD estimated at −0.09 (95% CI −0.19 to 0.00, *P*=.05). The LDL outcome (Figure 5) reported an SMD estimated at –0.18 (95% CI –0.33 to –0.04, *P*=.01). The TG outcome (Figure 6) reported an SMD estimated at −0.10 (95% CI –0.28 to 0.08), *P*=.28. Diastolic and systolic BP outcomes (Figures 7 and 8) reported SMDs estimated at –0.06 (95% CI −0.20 to 0.08, *P*=.43) and −0.03 (95% CI −0.18 to 0.13, *P*=.74), respectively. The HbA_1c_ outcome (Figure 9) reported an RR estimated at 1.04 (95% CI 0.40 to 2.70, *P*=.94). A summary of the clinical outcome findings is presented in Figures 2-9.

#### Behavioral Outcomes

**Figure 10:**
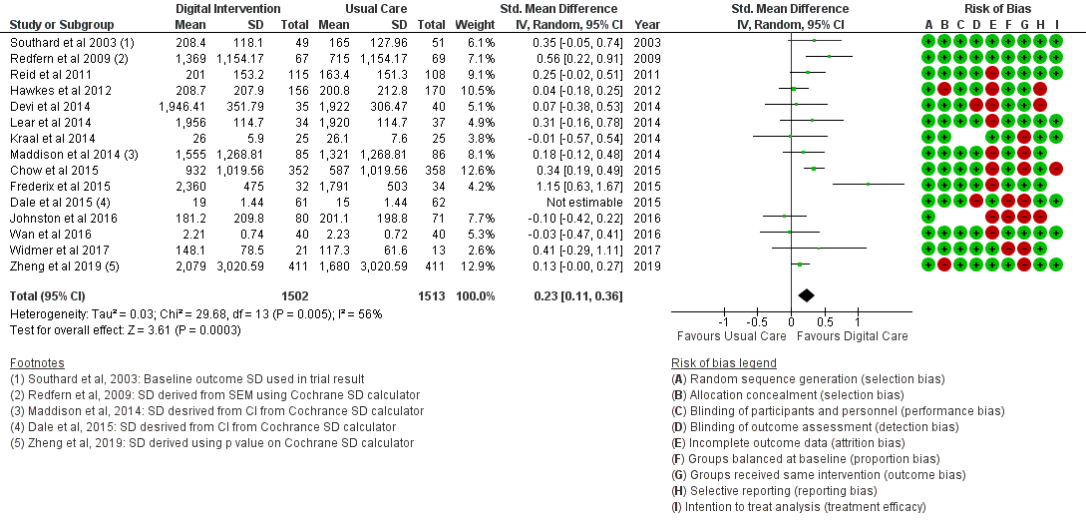
Outcomes of the examined studies for physical activity.

**Figure 11:**
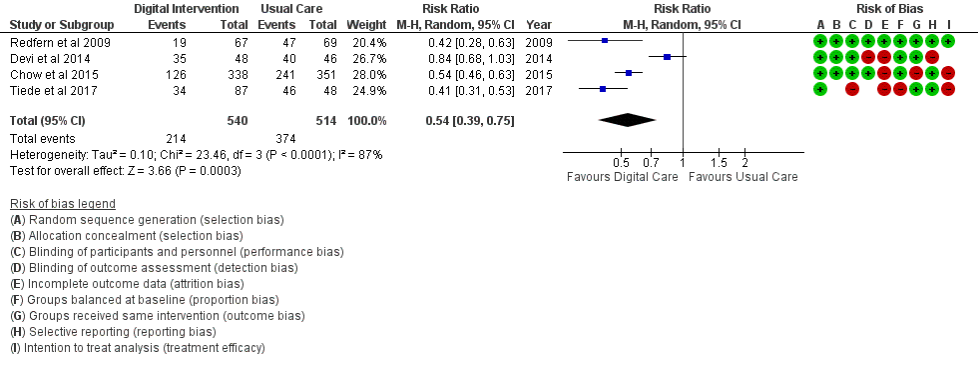
Outcomes of the examined studies for physical inactivity.

**Figure 12:**
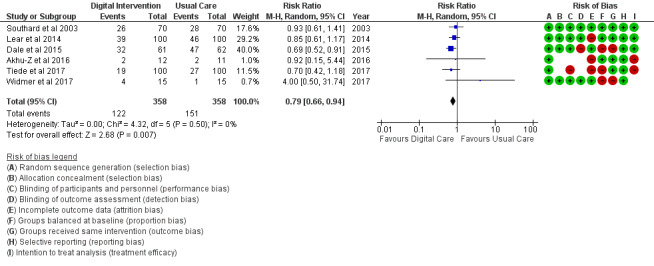
Outcomes of the examined studies for food intake.

**Figure 13:**
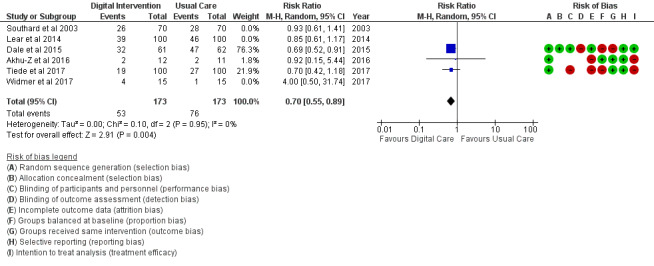
Outcomes of the examined studies for healthy diet.

**Figure 14:**
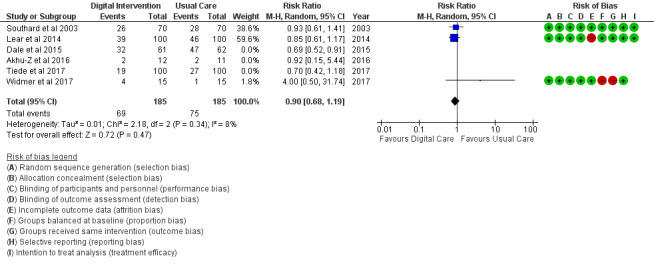
Outcomes of the examined studies for unhealthy food intake.

**Figure 15:**
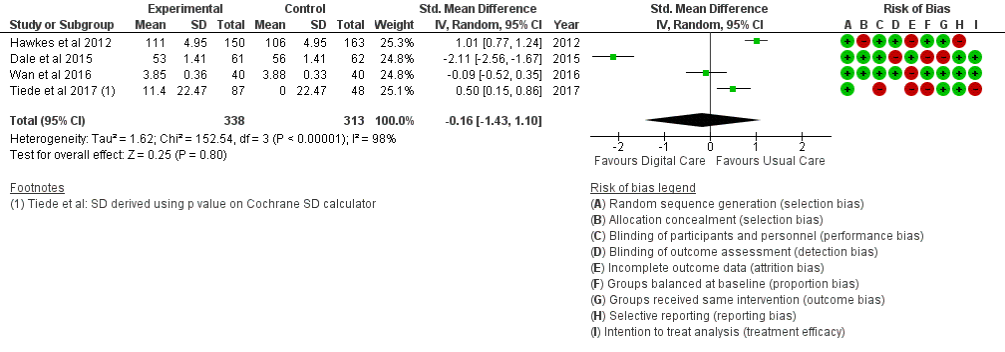
Outcomes of the examined studies for alcohol consumption.

**Figure 16:**
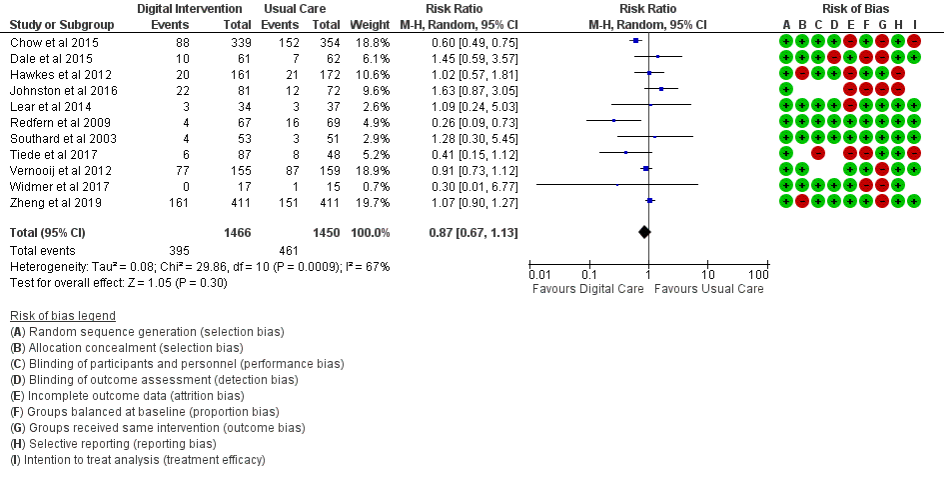
Outcomes of the examined studies for smoking.

**Figure 17:**
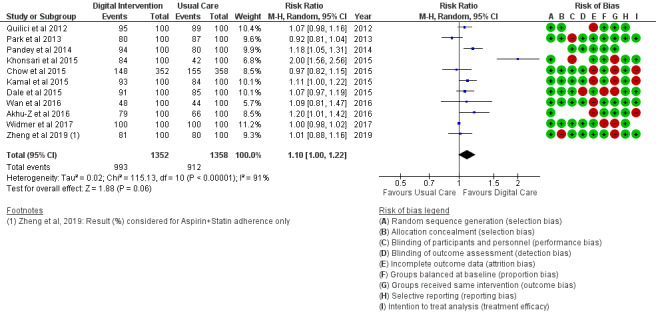
Medication adherence for all trials.

**Figure 18:**
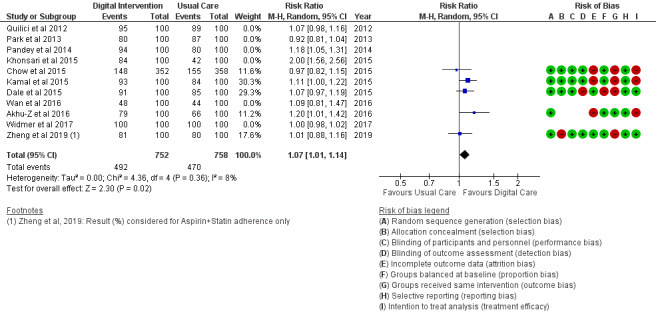
Medication adherence for multiple treatment with SMS.

**Figure 19:**
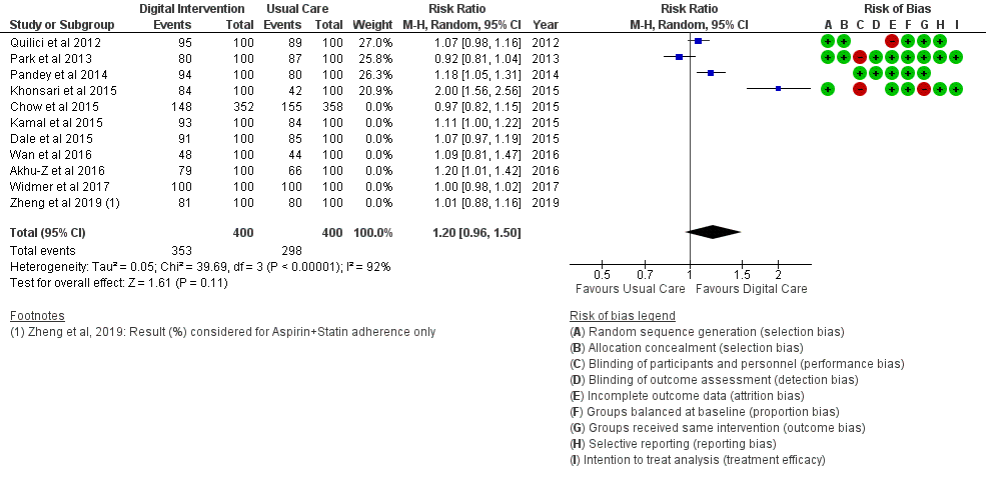
Medication adherence for target treatment only with SMS text message intervention.

**Figure 20:**
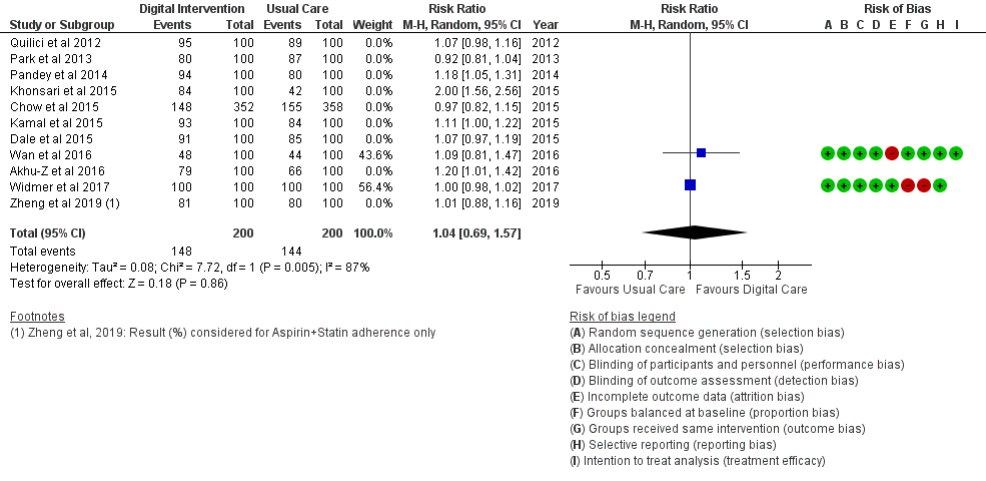
Medication adherence for treatment with non-sms intervention.

**Figure 21:**
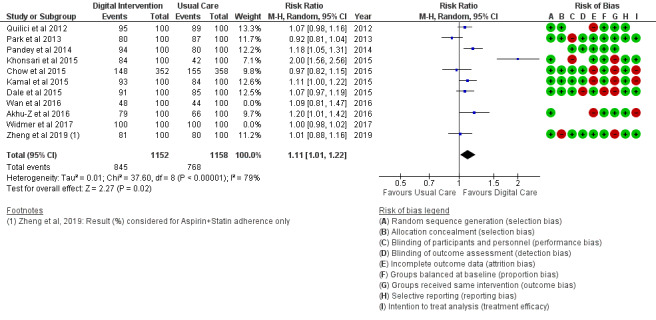
Medication adherence treatment for all sms intervention.

The PA outcome (Figure 10) reported an SMD estimated at 0.23 (95% CI 0.11 to 0.36, *P*<.001). PI (sedentary) in Figure 11 reported an RR estimated at 0.54 (95% CI 0.39 to 0.75, *P<*.001). Diet (food intake) in Figure 12 reported an RR estimated at 0.79 (0.66 to 0.94, *P*=.007). Further analysis was conducted as healthy diet targeted (Figure 13) treatment and unhealthy diet targeted (Figure 14) treatment; this is reported in the *Additional* *Analysis* section. The alcohol intake outcome (Figure 15) reported an SMD estimated at −0.16 (95% CI –1.43 to 1.10, *P*=.80). Smoking and medication adherence outcomes (Figures 16 and 17) reported RR estimated at 0.87 (95% CI 0.67 to 1.13, *P*=.30), and 1.10 (95% CI 1.00 to 1.22, *P*=.06), respectively. A summary of the behavioral outcome findings is presented in Figures 10-21.

### Risk of Bias in Included Studies

Table 2 provides a qualitative detail of the risk of bias assessment of the studies in the review. Proportion bias at baseline was reported in 16% (4/25) of the included studies as high risk. Intervention dropout was recorded in 32% (8/25) of the included studies at less than 10% of participants per study. Dropouts greater than 10% of study participants were recorded as high risk for treatment efficacy.

### Risk of Bias Across Studies

The results of the risk of bias across studies for each outcome are presented along with the meta-analysis (Figures 2-21). Outcomes are sparsely identified with low and unclear risks for the identified risk items.

### Additional Analysis

Results of the sensitivity analysis were included for the considered outcomes in Figures 13 and 14 and Figures 18-21. Sensitivity analyses proportionately conducted on outcomes to check the cumulative effects of the study population and intervention characteristics (eg, publication year, participant size, efficacy, and categories of treatment) on subgroups showed significant effects of interest in 2 outcomes as follows:

Food intake: Healthy diet targeted (Figure 13) treatment (*P*=.004) and unhealthy diet targeted (Figure 14) treatment (*P*=.47); medication adherence: medication adherence plus other risk factors (Figure 18) treatment (*P*=.02) and medication adherence treatment alone (*P*=.11), as shown in Figure 19.

## Discussion

### Principal Findings

This study of digital technology interventions addressing clinical and behavioral risk factor modification in people with CVDs demonstrates that not all CVD lifestyle risk factor modifications are favored by the use of digital interventions. Digital technology intervention in cardiac patients was associated with improvements in TC, HDL, LDL, PA, PI, healthy diet, and medication adherence (all *P*≤.05). However, there were no differences in intervention effects for BMI, TGs, BP (diastolic and systolic), blood sugar, alcohol intake, and smoking (all *P*>.05).

### Behavioral Change Constructs and Digital Intervention Strategies

The mechanism of risk factor modification in the included studies is based on behavioral change constructs [43,44], which include self-management, feedback mechanisms, progress recording and tracking (monitoring), one-on-one or social support, persuasion, personalization (customization), reiteration, self-efficacy, and motivation. For this study, these constructs are commonly used in the study trials compared with other constructs in the literature, such as perceived risk and perceived benefits, incentives, and reimbursement (rewards), which are rarely used in the study trials.

The use of behavioral change constructs in combination with digital technologies in the study trials has revealed their successful application in individual behavioral risk factor modification [7]. The overall desired effect has been found in digital interventions alone (15 studies) when compared with digital plus usual care interventions (10 studies), giving support to out-of-clinic risk factor modification at the personal level [9].

From our results, the use of behavioral change constructs and digital intervention strategies largely relies on patients’ self-dependency (low- to moderate-risk CVD patients), and interventions that favored digital technologies were reported for all CVD populations but barely for study trials on stroke and rarely for study trials on angina outpatients. An exception to this reliance was found in studies on medication adherence, which had been successfully self-managed with a digital intervention (SMS text messaging) - this informed advice-based instead of activity-based options for moderate-risk CVD outpatients in risk factor modification prescriptions. The effect of mobile sensor technology using wearable devices was inconclusive as there was only one study that engaged this digital intervention in its trial (Table S4 in Multimedia Appendix 1).

### Clinical Outcomes

There was no clinical benefit for BMI, TG, SBP, DBP, or HbA_1c_ with the use of digital interventions compared with usual care intervention in the study trials. This finding suggests a form of association between clinical factors and unhealthy behavior modification using digital technologies, noting that these clinical factors are the main indicators of unhealthy behavior conditions such as obesity, hypercholesterolemia, hypertension, and diabetes.

However, an exception to these similarities is the significant effect of digital technology intervention on clinical factors such as TC, HDL, and LDL, which could only be inferred by their shared physiological response to regulations in and by healthy behavioral factors such as healthy diet and medication adherence from a lifestyle perspective [45]. This response was less impactful on TGs, which are stored lipids in fatty cells and though considered bad cholesterol like LDL, are less regulated by medication (eg, statin) as compared with diet [45]. Our findings suggest that this exception is not necessarily based on the application of behavioral change techniques or other change-effecting variables. This view is validated by the fact that BMI (an indicator of overweight) and HbA_1c_ (an indicator of excess sugar), which are precursors for obesity and diabetes respectively as CVD risk factors, in association with unhealthy food intake appear to be not modifiable by digital technology interventions. Furthermore, we consider that the modification of LDL (bad cholesterol) by digital intervention might have been because of the positive inverse effect derived from the modification benefits of TC and HDL (good cholesterols) within each study population.

TC, HDL, and LDL modifications are associated with the use of cell phone devices in study trials. Behavior change techniques in TC, HDL, and LDL populations include self-reporting and self-recording of progress, one-on-one support, and persuasion. TC, HDL, and LDL study populations share commonly diagnosed CVDs and digital intervention strategies.

### Behavioral Outcomes

PA trials are characterized by smartphones and cell phone devices in a 1:1 ratio. In order of preference, the use of intervention strategies is, first, telerehabilitation and online education, followed by online feedback (tele-support) and telemonitoring, and finally, SMS text messaging support by active coaching (20% of trials). PI (sedentary) trials revealed a higher population mean age, which suggests a close association with comorbidity and immobility among outpatients [46]; therefore, there is a need to tailor digital intervention treatment to patients’ level of engagement. 

PA and PI have gained modification preferences and digital intervention effectiveness because of active participant engagement in 11 smartphone studies using tele-intervention (audiovisual) strategy, 2 cell phone studies engaging in active coaching (audio) strategy, and 6 cell phone studies using automated SMS text messaging support (text) strategy (in 19 studies). This finding suggests greater effectiveness of smartphones in audiovisual interventions compared with cell phones, indicating that cell phones might have gained usage (in medication adherence study trials) only because of their affordability and ease of use [44,47]. However, both audiovisual support and audio or text support appear to be efficient digital interventions for risk factor modification for PA; however, audio or text support only appears sufficient for PI modification.

Generally, PA (a healthy behavioral factor) has been viewed as a null to PI (sedentary; an unhealthy behavioral factor) effects in maintaining a healthy lifestyle. This view has been disapproved in the literature [48]. However, this disapproval has only been validated in a healthy population prospective study. More evidence is needed to validate this in a CVD population to elucidate the effectiveness of digital technology interventions for PI risk factor modification. Therefore, we consider that the modification of PI, just as in LDL, might have been because of the positive inverse effect of PA modification in the CVD populations reviewed.

Studies reporting effects on diet, alcohol consumption, and smoking share similar characteristics in behavioral change techniques, which include mostly social support and group discussion, followed by self-management, goal setting, follow-up, progress self-reporting and self-recording, and auto-reminders. Social support and group discussion, which are related to online support and online discussion, have been identified as activity-based behavioral change techniques in mental health management for diet, alcohol consumption, and smoking behavior modifications [49]. Interactivity (as a result of social support and group discussion) can, therefore, be affirmed as an effective factor in the behavior change technique of diet, alcohol consumption, and smoking on a digital platform. However, digital interventions for alcohol consumption and smoking behavior change show a weak effect in their modification when compared with conventional CVDs’ usual care interventions. There could be several reasons for this—first, social support and group discussions or interaction are less effectively accomplished compared with cell phone device interventions, which have no *smart* facial contact technology features but have gained wider usage in reviewed study trials because of their affordability and availability to participants in both risk factor studies. Second, digital technology interventions, from the trend seen in this study, appear to be effective in healthy behavior modification but less effective in attending to unhealthy behavior modification when compared with usual care: healthy dieting is physiologically linked to lipid regulation in the body [50], a strong basis for clinical factor (TC, HDL, and LDL) modification.

In addition, digital intervention effectiveness in TC, HDL, and LDL, as stated earlier, might also be largely linked to the positive pharmacological effect of medication adherence in study trials. Of the 6 studies on food intake (diet), unhealthy food intake (Figure 14) modification is not favored by digital intervention (*P*=.47); however, a healthy diet (Figure 13) shows a significant modification effect in favor of digital intervention (*P*=.004) when compared with usual care. This difference reveals significant alignment and potency of digital intervention toward healthy behavioral factors than unhealthy behavioral factors. The same was confirmed for PA, a healthy behavioral factor. Healthy behavioral factor (eg, PA, healthy diet, medication adherence) modification using digital technology is supported by findings from Chow et al [18].

Medication adherence outcome from trials in this study was achieved only by the use of cell phones with SMS text messaging support strategy, in line with the findings of Palmer et al [51]. However, the effectiveness of smartphones is inconclusive, as only 1 trial is available in this study—this could be responsible for its limitation in maximizing change technique features, for example, telerehabilitation in medication adherence trials. Cell phones remain the most affordable and available [47] digital devices in medication adherence-targeted interventions compared with other behavioral factor interventions as they cut across all CVD types and engage behavioral change techniques based on cognition such as auto-support, auto-reminders, persuasion (iteration), goal setting, self-management, and customization (personalization).

Trials (Figure 19) that strictly targeted medication adherence outcome only, using an SMS text messaging strategy with a cell phone device, did not show a significant effect (*P*=.11) when collectively analyzed for digital intervention effectiveness. However, trials (Figure 18) with a similar strategy and device as the former but having multiple clinical and behavioral outcome treatments (analyzed with or without the previous trials) were significantly effective (*P*=.02) with the use of digital intervention compared with usual care. Non-SMS-administered medication adherence trials (Figure 20) did not favor digital intervention. In summary, these findings suggest the effectiveness of multiple clinical and behavioral outcome treatments when designing digital technology (SMS text messaging) interventions. 

A few meta-analyzed results such as for smoking, LDL, BMI, and SBP were limited by high heterogeneity not fully explained (or not explained at all as for alcohol consumption and sedentary lifestyle with low included study counts) by study population or intervention characteristics. However, minor adjustments (exclusion of Chow et al [18], Widmer et al [8], and Redfern et al [33]) in the number of included studies toward increased homogeneity did not show a significant change from the initial treatment effect by either digital intervention or usual care.

The main intervention strategies in this study are automated SMS text messaging support (auto-reminder based on cognition which is largely accessible using cell phones in study trials), a feature supported by Kassavou et al [44]; and then online education and coaching, followed by telerehabilitation and telemonitoring, which were barely represented in analyses that favored digital intervention—representation might be because of limited access to smartphones based on the participants’ affordability or level of technological advancement or inclination at the time of the trial. A desirable device is the smartphone because it combines all operability features needed to attain desirable intervention outcomes by identifying behavioral change-specific strategies. However, a major limitation to the use of smartphones by the population age group in the study could be their level of comprehensibility [5].

### Limitations

Although this collection of studies is evenly distributed on a global scale, no RCT study has been identified in Africa, where only cost-effective digital health programs have presently gained widespread use [47]. A high proportion of male to female patients would be considered a major limitation of participant inclusion in studies. However, this trend appears to be in resonance with quantitative analyses of CVD gender prevalence in the literature [4] and, therefore, may reflect disease prevalence rather than study design.

This study further reveals gaps in the application of emerging technologies (immersive media, eg, 3D animations and games, an ongoing trial by Gallagher et al [52]; big data technologies, eg, artificial intelligence applications; and user experience) in CVD risk factor modification using evidence-based RCT intervention studies on a digital device platform. Therefore, this study suggests the initiation of cutting-edge research in the field of emerging digital technologies.

### Conclusions

This study shows that the use of digital technology interventions did not improve all CVD lifestyle risk factors compared with usual care interventions. Effective digital technology interventions appear to improve healthy behavioral factors (PA, healthy diet) and associated clinical outcomes (TC, HDL, and LDL), and were more potent in multiple outcome treatment (medication adherence plus) but were weak in abating unhealthy behavioral factors (smoking, alcohol intake, and unhealthy food intake) and their outcomes (BMI, BP, and HbA_1c_).

Cell phones are considered efficient digital devices for use with cognitive intervention strategies and have been most widely studied; however, smartphones may have advantages because of additional interaction features. This study was not able to analyze cutting-edge technology (such as immersive media technologies) as the data do not exist or are not reported. Newer immersive media technologies, therefore, warrant further study. Further RCT research is deemed necessary to consolidate the use of digital technology interventions, especially in CVD risk factors (eg, diabetes), with fewer RCT studies.

## Appendix

Multimedia Appendix 1 Protocol registration link and tables and abbreviations and definition of terms.

Multimedia Appendix 2 Study characteristics.

## Abbreviations

BP: blood pressure
CHD: coronary heart disease
CVD: cardiovascular disease
DBP: diastolic blood pressure
HbA_1c_: Hemoglobin A_1c_ blood sugar
HDL: high-density lipoprotein
LDL: low-density lipoprotein
MD: mean difference
PA: physical activity
PI: physical inactivity
PRISMA: Preferred Reporting Items for Systematic Reviews and Meta-analysis
RCT: randomized controlled trial
RR: risk ratio
SBP: systolic blood pressure
SMD: standard mean difference
TC: total cholesterol
TG: triglyceride
