# Supplementary material for: Digital Technology Interventions for Risk Factor Modification in Patients With Cardiovascular Disease: Systematic Review and Meta-analysis
Source: JMIR Mhealth Uhealth. 2021 Mar 3;9(3):e21061. doi: 10.2196/21061 (PMC7970167; doi:10.2196/21061)
Supplement: Multimedia Appendix 1 [file mhealth_v9i3e21061_app1.doc]

**Multimedia Appendix 1.** Protocol registration link and tables and abbreviations and definition of terms.

**Protocol Registration: PROSPERO**

**Link:** <https://www.crd.york.ac.uk/prospero/display_record.php?ID=CRD42019139801>

**Table 3: Table of search log template**

|  | **Medline (Ovid)**  **25.04-09.2019** |  |  |
| --- | --- | --- | --- |
| **#** | **Searches** | **Results** | **Type** |
| 1 | Telemedicine/ | 19261 | Advanced |
| 2 | exp Telerehabilitation/ | 240 | Advanced |
| 3 | 1 or 2 | 19489 | Advanced |
| 4 | (digital technolog* or electronic technolog* or mobile technolog*).af. | 4472 | Advanced |
| 5 | (digital devic* or electronic devic* or mobile devic*).af. | 11475 | Advanced |
| 6 | (monitor* devic* or monitor* technolog* or monitor* app*).af. | 6514 | Advanced |
| 7 | (telehealth or tele-health).af. | 4441 | Advanced |
| 8 | (e-health or ehealth or electronic health).af. | 33701 | Advanced |
| 9 | (m-health or mhealth or mobile health).af. | 15482 | Advanced |
| 10 | (telemedicine or tele-medicine).af. | 26720 | Advanced |
| 11 | exp Mobile Applications/ | 3997 | Advanced |
| 12 | (mobile app* or digital app* or electronic app*).af. | 7640 | Advanced |
| 13 | (emerging technolog* or emergent technolog* or emerging digital technolog* or emergent digital technolog*).af. | 5804 | Advanced |
| 14 | (artificial* intelligen* or AI or machine learn* or ML or deep learn* or DL or blockchain or IoT or internet of things).af. | 1686690 | Advanced |
| 15 | (immersive media or augment* realit* or AR or virtual realit* or VR or mixed realit* or MR or digital screen*).af. | 334775 | Advanced |
| 16 | (predict* analy* or robot* or fog comput* or nanotech* or cloud comput* or bionics).af. | 173066 | Advanced |
| 17 | (5G technolog* or fifth generat* technolog* or 5G network* or fifth generat* network* or 5G broadband or 5G cellular* or fifth generat* cellular or fifth generat* broadband).af. | 42 | Advanced |
| 18 | (3D or 3-D or three dimens* or 3-dimens*).af. | 346512 | Advanced |
| 19 | (CX or customer* experienc*).af. | 15059 | Advanced |
| 20 | Technology, High-Cost/ | 1206 | Advanced |
| 21 | digital health.af. | 1476 | Advanced |
| 22 | or/1-21 | 2549482 | Advanced |
| 23 | exp Cardiovascular Diseases/ | 2265537 | Advanced |
| 24 | cardiovascular diseas*.af. | 270561 | Advanced |
| 25 | coronary arter* diseas*.af. | 116099 | Advanced |
| 26 | (stroke or strokes or cerebral vascular accident*).af. | 354374 | Advanced |
| 27 | peripheral arter* diseas*.af. | 15445 | Advanced |
| 28 | atherosclerosis.af. | 185542 | Advanced |
| 29 | (atrial fibrillation or Afib).af. | 74261 | Advanced |
| 30 | (myocardial infarction* or heart attack* or myocardial ischem*).af. | 279301 | Advanced |
| 31 | kidney arter* diseas*.af. | 0 | Advanced |
| 32 | exp Renal Artery Obstruction/ | 10166 | Advanced |
| 33 | (Renal Arter* diseas* or Renal Arter* Obstruct* or Renal Arter* stenosis).af. | 11949 | Advanced |
| 34 | or/23-33 | 2573144 | Advanced |
| 35 | exp Health Behavior/ | 295076 | Advanced |
| 36 | (behav* chang* or Chang* behav*).af. | 34751 | Advanced |
| 37 | (behav* mod* or mod* behav*).af. | 18728 | Advanced |
| 38 | illness belie*.af. | 460 | Advanced |
| 39 | (health* lifestyl* or health* life styl*).af. | 9822 | Advanced |
| 40 | exp attitude to health/ | 389076 | Advanced |
| 41 | (Attitud* to Health or health* attitud*).af. | 274330 | Advanced |
| 42 | or/35-41 | 584425 | Advanced |
| 43 | exp risk factors/ | 764669 | Advanced |
| 44 | (risk* factor* and health).af. | 366171 | Advanced |
| 45 | exposure factor*.af. | 950 | Advanced |
| 46 | protect* factor*.af. | 20593 | Advanced |
| 47 | exp Protective Factors/ | 3116 | Advanced |
| 48 | exp Risk-Taking/ | 29947 | Advanced |
| 49 | (risk manifest* or manifest* risk).af. | 141 | Advanced |
| 50 | (risk trigger* or trigger* risk*).af. | 58 | Advanced |
| 51 | (health outcome* or outcome* health).af. | 49419 | Advanced |
| 52 | (health hazard* or hazard* health).af. | 8678 | Advanced |
| 53 | (exposure risk* or risk* exposur*).af. | 3567 | Advanced |
| 54 | (behav* risk* or risk* behav*).af. | 40273 | Advanced |
| 55 | or/43-54 | 963371 | Advanced |
| 56 | digital*.af. | 148395 | Advanced |
| 57 | (health interven* or interven* health).af. | 13130 | Advanced |
| 58 | 56 and 57 | 295 | Advanced |
| 59 | exp health promotion/ or exp health education/ | 232011 | Advanced |
| 60 | (digital* rehab* or digital* re-hab*or re-hab* digital* or rehab* digital*).af. | 15 | Advanced |
| 61 | (health* network* or network* health* or (social* intereact* or interact* social*)).af. | 31344 | Advanced |
| 62 | cessation program*.af. | 3108 | Advanced |
| 63 | exp community participation/ | 39711 | Advanced |
| 64 | (communit* engag* or engag* communit* or communit* participat*).af. | 25321 | Advanced |
| 65 | exp self care/ or exp social networking/ | 54335 | Advanced |
| 66 | (self-manag* or self-efficac* or self care).af. | 80070 | Advanced |
| 67 | (intervent* health* polic* or health* intervention* polic*).af. | 34 | Advanced |
| 68 | secondary prevention.af. | 33357 | Advanced |
| 69 | (risk factor modification or risk factor management).af. | 2084 | Advanced |
| 70 | exp Self-Help Groups/ or exp Social Support/ | 74526 | Advanced |
| 71 | or/56-69 | 576274 | Advanced |
| 72 | exp rural health services/ or exp rural population/ or exp rural health/ or exp hospitals, rural/ or exp rural nursing/ | 89275 | Advanced |
| 73 | (remot* or rural*).af. | 244435 | Advanced |
| 74 | or/72-73 | 244435 | Advanced |
| 75 | 22 and 34 and 42 and 55 and 71 | 317 | Advanced |
| 76 | 22 and 34 and 42 and 55 and 71 and 74 | 28 | Advanced |
| 77 | limit 75 to english language | 305 | Advanced |
| 78 | limit 77 to meta analysis | 9 | Advanced |
| 79 | limit 77 to ("systematic review" or systematic reviews as topic) | 10 | Advanced |
| 80 | (metaanal* or meta-anal* or systematic).ti. | 178907 | Advanced |
| 81 | 77 and 80 | 11 | Advanced |
| 82 | 78 or 79 or 81 | 15 | Advanced |

|  | **COCHRANE Library** |  |
| --- | --- | --- |
|  | **01/05/2019 20:55** |  |
|  | Mendeley Ref.: 129 Reviews but 128 imported. 84 Trials but 76 imported. 1 Protocol. |  |
| ID | Search | Hits |
| #1 | MeSH descriptor: [Rural Health Services] explode all trees | 329 |
| #2 | MeSH descriptor: [Rural Population] explode all trees | 1513 |
| #3 | MeSH descriptor: [Rural Health] explode all trees | 520 |
| #4 | MeSH descriptor: [Hospitals, Rural] explode all trees | 34 |
| #5 | MeSH descriptor: [Rural Nursing] explode all trees | 1 |
| #6 | “remot*” or “rural*” | 8997 |
| #7 | {OR #1-#6} | 8997 |
| #8 | digital* | 10976 |
| #9 | “health intervent*” or “intervent* health” | 1 |
| #10 | #8 and #9 | 0 |
| #11 | MeSH descriptor: [Health Promotion] explode all trees | 5720 |
| #12 | MeSH descriptor: [Health Education] explode all trees | 18162 |
| #13 | “digital* rehab*” or “digital* re-hab*” or “re-hab* digital*” or “rehab* digital*” | 0 |
| #14 | “health* network*” or “network* health*” or “social* intereact*” or “interact* social*” | 926 |
| #15 | cessation program* | 3970 |
| #16 | MeSH descriptor: [Community Participation] explode all trees | 1452 |
| #17 | “communit* engag*” or “engag* communit*” or “communit* participat*” | 0 |
| #18 | MeSH descriptor: [Self Care] explode all trees | 5245 |
| #19 | MeSH descriptor: [undefined] explode all trees | 0 |
| #20 | MeSH descriptor: [Self Efficacy] explode all trees | 2779 |
| #21 | MeSH descriptor: [Self-Management] explode all trees | 177 |
| #22 | MeSH descriptor: [Blood Glucose Self-Monitoring] explode all trees | 679 |
| #23 | “self-manag*” or “self-efficac*” or “self care” | 9448 |
| #24 | “intervent* health* polic*” or “health* intervention* polic*” | 0 |
| #25 | MeSH descriptor: [Health Policy] explode all trees | 572 |
| #26 | MeSH descriptor: [Secondary Prevention] explode all trees | 2964 |
| #27 | “risk factor modification” or “risk factor management” | 504 |
| #28 | MeSH descriptor: [Social Support] explode all trees | 3133 |
| #29 | MeSH descriptor: [Self-Help Groups] explode all trees | 739 |
| #30 | {OR #8-#29} | 49708 |
| #31 | MeSH descriptor: [Risk Factors] explode all trees | 23777 |
| #32 | “risk* factor* and health” | 1 |
| #33 | exposure factor* | 4 |
| #34 | MeSH descriptor: [Protective Factors] explode all trees | 83 |
| #35 | protect* factor* | 3 |
| #36 | MeSH descriptor: [Risk-Taking] explode all trees | 1480 |
| #37 | “risk manifest*” or “manifest* risk” | 3 |
| #38 | “risk trigger*” or “trigger* risk*” | 3 |
| #39 | “health outcome*” or “outcome* health” | 1673 |
| #40 | “health hazard*” or “hazard* health” | 514 |
| #41 | “exposure risk*” or “risk* exposur*” | 124 |
| #42 | “behav* risk*” or “risk* behav*” | 0 |
| #43 | {OR #31-#42} | 27392 |
| #44 | MeSH descriptor: [Health Behavior] explode all trees | 33094 |
| #45 | (behav* chang*) or (chang* behav*) | 39151 |
| #46 | (behav* mod*) or (mod* behav*) | 46164 |
| #47 | illness belie* | 2115 |
| #48 | (health* lifestyl*) or (health* life styl*). | 13079 |
| #49 | MeSH descriptor: [Attitude to Health] explode all trees | 33125 |
| #50 | “behav* chang*” or “chang* behav*” | 1 |
| #51 | {OR #44-#50} | 104658 |
| #52 | MeSH descriptor: [Cardiovascular Diseases] explode all trees | 96445 |
| #53 | cardiovascular diseas* | 49724 |
| #54 | coronary arter* diseas* | 23020 |
| #55 | “stroke" or "strokes” or “cerebral vascular accident*” | 63036 |
| #56 | peripheral arter* diseas* | 6590 |
| #57 | MeSH descriptor: [Atherosclerosis] explode all trees | 1855 |
| #58 | “atrial fibrillation” or “Afib” | 11434 |
| #59 | “myocardial infarction*” or “heart attack*” or “heart failure” or “myocardial ischem*” | 28962 |
| #60 | MeSH descriptor: [Atrial Fibrillation] explode all trees | 3951 |
| #61 | MeSH descriptor: [Stroke] explode all trees | 8172 |
| #62 | kidney arter* diseas* | 3776 |
| #63 | MeSH descriptor: [Renal Artery Obstruction] explode all trees | 124 |
| #64 | “Renal Arter* diseas*” or “Renal Arter* Obstruct*” or “Renal Arter* stenosis” | 0 |
| #65 | {OR #52-#64} | 189887 |
| #66 | MeSH descriptor: [Telemedicine] explode all trees | 2072 |
| #67 | “digital technolog*” or “electronic technolog*” or “mobile technolog*” | 2 |
| #68 | “digital devic*” or “electronic devic*” or “mobile devic*” | 20 |
| #69 | “monitor* devic*” or “monitor* technolog*” or “monitor* app*” | 5 |
| #70 | “telehealth” or “tele-health” | 1174 |
| #71 | “e-health” or “ehealth” or “electronic health” | 6855 |
| #72 | “m-health” or “mhealth” or “mobile health” | 5193 |
| #73 | “telemedicine” or “tele-medicine” | 3250 |
| #74 | MeSH descriptor: [Mobile Applications] explode all trees | 380 |
| #75 | “emerging technolog*” or “emergent technolog*” or “emerging digital technolog*” or “emergent digital technolog*” | 0 |
| #76 | “artificial* intelligen*” or “AI” or “machine learn*” or “ML” or “deep learn*” or “DL” or “blockchain” or “IoT” or “internet of things” | 131361 |
| #77 | “immersive media” or “augment* realit*” or “AR” or “virtual realit*” or “VR” or “mixed realit*” or “MR” or “digital screen*” | 39264 |
| #78 | “predict* analy*” or “robot*” or “fog comput*” or “nanotech*” or “cloud comput*” or “bionics” | 2189 |
| #79 | “5G technolog*” or “fifth generat* technolog*” or “5G network*” or “fifth generat* network*” or “5G broadband” or “5G cellular*” or “fifth generat* cellular” or “fifth generat* broadband” | 0 |
| #80 | “3D” or “three dimens*” or “3 dimens*” | 4713 |
| #81 | “CX” or “customer* experienc*” | 1167 |
| #82 | MeSH descriptor: [Technology, High-Cost] explode all trees | 3 |
| #83 | digital health | 137 |
| #84 | MeSH descriptor: [Internet] explode all trees |  |
| #85 | {OR #66-#84} | 183051 |
| #86 | #30 and #43 and #51 and #65 and #84 | 234 |
| #87 | #7 and #30 and #43 and #51 and #65 and #84 | 45 |

|  | **CINAHL Complete** |  |
| --- | --- | --- |
|  | **01.05.2019. 15:43** |  |
|  | Comments: |  |
| # | Search | Results |
| 1 | (MH "Telemedicine+") OR (MH "Telerehabilitation") OR (MH "Telehealth+") | 18,774 |
| 2 | digital technolog* or electronic technolog* or mobile technolog* | 3,422 |
| 3 | digital devic* or electronic devic* or mobile devic* | 3,674 |
| 4 | monitor* devic* or monitor* technolog* or monitor* app* | 4,434 |
| 5 | e-health or ehealth or electronic health | 28,343 |
| 6 | m-health or mhealth or mobile health | 4,208 |
| 7 | telemedicine or tele-medicine | 10,442 |
| 8 | telehealth or tele-health | 7,897 |
| 9 | (MH "Mobile Applications") | 5,207 |
| 10 | mobile app* or digital app* or electronic app* | 8,198 |
| 11 | emerging technolog* or emergent technolog* or emerging digital technolog* or emergent digital technolog* | 1,818 |
| 12 | artificial* intelligen* or AI or machine learn* or ML or deep learn* or DL or blockchain or IoT or internet of things | 84,076 |
| 13 | immersive media or augment* realit* or virtual realit* or mixed realit* or digital screen* | 6,211 |
| 14 | predict* analy* or robot* or fog comput* or nanotech* or cloud comput* or bionics | 21,404 |
| 15 | 5G technolog* or fifth generat* technolog* or 5G network* or fifth generat* network* or 5G broadband or 5G cellular* or fifth generat* cellular or fifth generat* broadband | 7 |
| 16 | 3D or 3-D or three dimens* or 3-dimens* | 35,885 |
| 17 | CX or customer* experienc* | 423 |
| 18 | (MH "Health Care Costs+") | 49,777 |
| 19 | digital health | 945 |
| 20 | S1 OR S2 OR S3 OR S4 OR S5 OR S6 OR S7 OR S8 OR S9 OR S10 OR S11 OR S12 OR S13 OR S14 OR S15 OR S16 OR S17 OR S18 OR S19 | 256,972 |
| 21 | (MH "Cardiovascular Diseases+") | 496,506 |
| 22 | coronary arter* diseas* | 17,347 |
| 23 | stroke or strokes or cerebral vascular accident* | 100,760 |
| 24 | peripheral arter* diseas* | 3,850 |
| 25 | (MH "Atherosclerosis") | 7,380 |
| 26 | atrial fibrillation or Afib | 28,041 |
| 27 | myocardial infarction* or heart attack* or myocardial ischem* | 61,844 |
| 28 | kidney arter* diseas* | 64 |
| 29 | (MH "Renal Artery Obstruction") | 807 |
| 30 | Renal Arter* diseas* or Renal Arter* Obstruct* or Renal Arter* stenosis | 1,136 |
| 31 | S21 OR S22 OR S23 OR S24 OR S25 OR S26 OR S27 OR S28 OR S29 OR S30 | 533,965 |
| 32 | (MH "Health Behavior+") | 87,533 |
| 33 | behav* chang* or Chang* behav* | 28,373 |
| 34 | behav* mod* or mod* behav* | 18,159 |
| 35 | illness belie* | 675 |
| 36 | health* lifestyl* or health* life styl* | 7,777 |
| 37 | (MH "Attitude to Health+") OR (MH "Attitude to Illness+") | 154,367 |
| 38 | S32 OR S33 OR S34 OR S35 OR S36 OR S37 | 227,552 |
| 39 | (MH "Cardiovascular Risk Factors") OR (MH "Risk Factors+") | 174,065 |
| 40 | risk* factor* and health | 115,260 |
| 41 | exposure factor* | 3,179 |
| 42 | protect* factor* | 6,927 |
| 43 | (MH "Risk Taking Behavior+") | 18,598 |
| 44 | risk manifest* or manifest* risk | 734 |
| 45 | risk trigger* or trigger* risk* | 397 |
| 46 | health outcome* or outcome* health | 83,361 |
| 47 | health hazard* or hazard* health | 2,716 |
| 48 | exposure risk* or risk* exposur* | 10,772 |
| 49 | behav* risk* or risk* behav* | 35,734 |
| 50 | S39 OR S40 OR S41 OR S42 OR S43 OR S44 OR S45 OR S46 OR S47 OR S48 OR S49 | 353,837 |
| 51 | digital | 32,121 |
| 52 | health interven* or interven* health | 26,340 |
| 53 | S51 AND S52 | 292 |
| 54 | (MH "Health Promotion+") | 58,249 |
| 55 | (MH "Health Education+") | 114,946 |
| 56 | digital* rehab* or digital* re-hab*or re-hab* digital* or rehab* digital* | 55 |
| 57 | (MH "Smoking Cessation Programs") | 2,061 |
| 58 | health* network* or network* health* or social* intereact* or interact* social* | 10,459 |
| 59 | communit* engag* or engag* communit* or communit* participat* | 8,939 |
| 60 | (MH "Self Care+") | 43,623 |
| 61 | (MH "Social Networking+") | 2,134 |
| 62 | (MH "Self-Management") | 180 |
| 63 | (MH "Self-Efficacy") | 18,128 |
| 64 | intervent* health* polic* or health* intervention* polic* | 372 |
| 65 | secondary prevention | 5,959 |
| 66 | risk factor modif* or risk factor manag* | 2,367 |
| 67 | (MH "Support Groups+") | 10,191 |
| 68 | Social Support | 25,200 |
| 69 | S51 OR S52 OR S53 OR S54 OR S55 OR S56 OR S57 OR S58 OR S59 OR S60 OR S61 OR S62 OR S63 OR S64 OR S65 OR S66 OR S67 OR S68 | 317,798 |
| 70 | (MH "Rural Population") OR (MH "Rural Health Centers") OR (MH "Hospitals, Rural") OR (MH "Rural Health Services") OR (MH "Rural Health Personnel") | 17,192 |
| 71 | (MH "Rural Health") OR (MH "Rural Health Nursing") | 8,113 |
| 72 | rural or remote | 69,877 |
| 73 | S70 OR S71 OR S72 | 69,877 |
| 74 | S20 AND S31 AND S38 AND S50 AND S69 AND S73 | 16 |
| 75 | S20 AND S31 AND S38 AND S50 AND S69 | 172 |
| 76 | S20 AND S31 AND S38 AND S50 AND S69 – narrowed, English. | 142 |

|  | **Web of science** |  |
| --- | --- | --- |
|  | **(07.05.2019 10.41)** |  |
| # | Search | Results |
| S1 | TS=("Telemedicine" OR "Telerehabilitation" OR "Telehealth" OR "digital technolog*" OR "electronic technolog*" OR "mobile technolog*" OR "digital devic*" OR "electronic devic*" OR "mobile devic*" OR "monitor* devic*" OR "monitor* technolog*" OR "monitor* app*" OR e-health OR ehealth OR "electronic health" OR m-health OR mhealth OR "mobile health" OR "mobile app*" OR "digital app*" OR "electronic app*" OR "emerging technolog*" OR "emergent technolog*" OR "emerging digital technolog*" OR "emergent digital technolog*" OR "artificial* intelligen*" OR ai OR "machine learn*" OR ml OR "deep learn*" OR dl OR "blockchain" OR iot OR "internet of things" OR "immersive media" OR "augment* realit*" OR ar OR "virtual realit*" OR vr OR "mixed realit*" OR mr OR "digital screen*" OR "predict* analy*" OR robot* OR "fog comput*" OR nanotech* OR "cloud comput*" OR bionics OR "5G technolog*" OR "fifth generat* technolog*" OR "5G network*" OR "fifth generat* network*" OR "5G broadband" OR "5G cellular*" OR "fifth generat* cellular" OR "fifth generat* broadband" OR 3d OR 3-d OR "three dimens*" OR 3-dimens* OR cx OR "customer* experienc*" OR "Technology, High-Cost" OR "digital health" OR “Internet”) | 2,896,575 |
| S2 | TS=("Renal Arter* diseas*" OR "Renal Arter* Obstruct*" OR "Renal Arter* stenosis" OR "Renal Artery Obstruction" OR "kidney arter* diseas*" OR "myocardial infarction*" OR "heart attack*" OR "heart failure*" OR "myocardial ischem*" OR "atrial fibrillation" OR afib OR atherosclerosis OR "peripheral arter* diseas*" OR stroke OR strokes OR "cerebral vascular accident*" OR "coronary arter* diseas*" OR "cardiovascular diseas*") | 1,018,507 |
| S3 | TS=("Attitud* to Health" OR "health* attitud*" OR "health* lifestyl*" OR "health* life styl*" OR "illness belie*" OR "behav* mod*" OR "mod* behav*" OR "behav* chang*" OR "Chang* behav*" OR "Health Behavi*") | 111,330 |
| S4 | TS=("behav* risk*" OR "risk* behav*" OR "exposure risk*" OR "risk* exposur*" OR "health hazard*" OR "hazard* health" OR "health outcome*" OR "outcome* health" OR "risk trigger*" OR "trigger* risk*" OR "risk manifest*" OR "manifest* risk" OR risk-taking OR "protect* factor*" OR "exposure factor*" OR "risk* factor*") | 842,845 |
| S5 | TS=("Self-Help Groups" OR "Social Support" OR "risk factor modification" OR "risk factor management" OR "secondary prevention" OR "intervent* health* polic*" OR "health* intervention* polic*" OR "self-manag*" OR "self-efficac*" OR "self care" OR "self care" OR "social networking" OR "communit* engag*" OR "engag* communit*" OR "communit* participat*" OR "community participation" OR "cessation program*" OR "health* network*" OR "network* health*" OR "social* interact*" OR "interact* social*" OR "digital* rehab*" OR "digital* re-hab*" OR "re-hab* digital*" OR "rehab* digital*" OR "health promotion" OR "health education" OR ("digital*" AND ("health interven*" OR "interven* health"))) | 289,910 |
| S6 | TS=("rural health services" OR "rural population" OR "rural health" OR "rural hospitals" OR "rural nursing" OR "remot*” or “rural*") | 523,680 |
| S7 | #6 AND #5 AND #4 AND #3 AND #2 AND #1 | 11 |
| S8 | #5 AND #4 AND #3 AND #2 AND #1 | 68 |
| S9 | (#5 AND #4 AND #3 AND #2 AND #1) *AND*LANGUAGE: (English) | 79 |

|  | **Scopus** |  |
| --- | --- | --- |
|  | **(13.05.2019 10.35)** |  |
| # | Search | Results |
| 1 | ( TITLE-ABS-KEY ( "Telemedicine" OR "Telerehabilitation" OR "Telehealth" OR "digital technolog*" OR "electronic technolog*" OR "mobile technolog*" OR "digital devic*" OR "electronic devic*" OR "mobile devic*" OR "monitor* devic*" OR "monitor* technolog*" OR "monitor* app*" OR e-health OR ehealth OR "electronic health" OR m-health OR mhealth OR "mobile health" OR "mobile app*" OR "digital app*" OR "electronic app*" OR "emerging technolog*" OR "emergent technolog*" OR "emerging digital technolog*" OR "emergent digital technolog*" OR "artificial* intelligen*" OR ai OR "machine learn*" OR ml OR "deep learn*" OR dl OR "blockchain" OR iot OR "internet of things" OR "immersive media" OR "augment* realit*" OR ar OR "virtual realit*" OR vr OR "mixed realit*" OR mr OR "digital screen*" OR "predict* analy*" OR robot* OR "fog comput*" OR nanotech* OR "cloud comput*" OR bionics OR "5G technolog*" OR "fifth generat* technolog*" OR "5G network*" OR "fifth generat* network*" OR "5G broadband" OR "5G cellular*" OR "fifth generat* cellular" OR "fifth generat* broadband" OR 3d OR 3-d OR "three dimens*" OR 3-dimens* OR cx OR "customer* experienc*" OR "Technology, High-Cost" OR "digital health" OR “Internet”) ) | 4,451,889 |
| 2 | ( TITLE-ABS-KEY ( "Renal Arter* diseas*" OR "Renal Arter* Obstruct*" OR "Renal Arter* stenosis" OR "Renal Artery Obstruction" OR "kidney arter* diseas*" OR "myocardial infarction*" OR "heart attack*" OR "heart failure*" OR "myocardial ischem*" OR "atrial fibrillation" OR afib OR atherosclerosis OR "peripheral arter* diseas*" OR stroke OR strokes OR "cerebral vascular accident*" OR "coronary arter* diseas*" OR "cardiovascular diseas*" ) ) | 1,304,997 |
| 3 | ( TITLE-ABS-KEY ( "Attitud* to Health" OR "health* attitud*" OR "health* lifestyl*" OR "health* life styl*" OR "illness belie*" OR "behav* mod*" OR "mod* behav*" OR "behav* chang*" OR "Chang* behav*" OR "Health Behavi*" ) ) | 340,889 |
| 4 | ( TITLE-ABS-KEY ( "behav* risk*" OR "risk* behav*" OR "exposure risk*" OR "risk* exposur*" OR "health hazard*" OR "hazard* health" OR "health outcome*" OR "outcome* health" OR "risk trigger*" OR "trigger* risk*" OR "risk manifest*" OR "manifest* risk" OR risk-taking OR "protect* factor*" OR "exposure factor*" OR "risk* factor*" ) ) | 1,499,863 |
| 5 | ( TITLE-ABS-KEY ( "Self-Help Groups" OR "Social Support" OR "risk factor modification" OR "risk factor management" OR "secondary prevention" OR "intervent* health* polic*" OR "health* intervention* polic*" OR "self-manag*" OR "self-efficac*" OR "self care" OR "self care" OR "social networking" OR "communit* engag*" OR "engag* communit*" OR "communit* participat*" OR "community participation" OR "cessation program*" OR "health* network*" OR "network* health*" OR "social* interact*" OR "interact* social*" OR "digital* rehab*" OR "digital* re-hab*" OR "re-hab* digital*" OR "rehab* digital*" OR "health promotion" OR "health education" OR ( "digital*" AND ( "health interven*" OR "interven* health" ) ) ) ) | 671,620 |
| 6 | ( TITLE-ABS-KEY ( "rural health services" OR "rural population" OR "rural health" OR "rural hospitals" OR "rural nursing" OR "remot*" OR "rural*" ) ) | 839,870 |
| 7 | ( ( TITLE-ABS-KEY ( "Telemedicine" OR "Telerehabilitation" OR "Telehealth" OR "digital technolog*" OR "electronic technolog*" OR "mobile technolog*" OR "digital devic*" OR "electronic devic*" OR "mobile devic*" OR "monitor* devic*" OR "monitor* technolog*" OR "monitor* app*" OR e-health OR ehealth OR "electronic health" OR m-health OR mhealth OR "mobile health" OR "mobile app*" OR "digital app*" OR "electronic app*" OR "emerging technolog*" OR "emergent technolog*" OR "emerging digital technolog*" OR "emergent digital technolog*" OR "artificial* intelligen*" OR ai OR "machine learn*" OR ml OR "deep learn*" OR dl OR "blockchain" OR iot OR "internet of things" OR "immersive media" OR "augment* realit*" OR ar OR "virtual realit*" OR vr OR "mixed realit*" OR mr OR "digital screen*" OR "predict* analy*" OR robot* OR "fog comput*" OR nanotech* OR "cloud comput*" OR bionics OR "5G technolog*" OR "fifth generat* technolog*" OR "5G network*" OR "fifth generat* network*" OR "5G broadband" OR "5G cellular*" OR "fifth generat* cellular" OR "fifth generat* broadband" OR 3d OR 3-d OR "three dimens*" OR 3-dimens* OR cx OR "customer* experienc*" OR "Technology, High-Cost" OR "digital health" ) ) ) AND ( ( TITLE-ABS-KEY ( "Renal Arter* diseas*" OR "Renal Arter* Obstruct*" OR "Renal Arter* stenosis" OR "Renal Artery Obstruction" OR "kidney arter* diseas*" OR "myocardial infarction*" OR "heart attack*" OR "myocardial ischem*" OR "atrial fibrillation" OR afib OR atherosclerosis OR "peripheral arter* diseas*" OR stroke OR strokes OR "cerebral vascular accident*" OR "coronary arter* diseas*" OR "cardiovascular diseas*" ) ) ) AND ( ( TITLE-ABS-KEY ( "Attitud* to Health" OR "health* attitud*" OR "health* lifestyl*" OR "health* life styl*" OR "illness belie*" OR "behav* mod*" OR "mod* behav*" OR "behav* chang*" OR "Chang* behav*" OR "Health Behavi*" ) ) ) AND ( ( TITLE-ABS-KEY ( "behav* risk*" OR "risk* behav*" OR "exposure risk*" OR "risk* exposur*" OR "health hazard*" OR "hazard* health" OR "health outcome*" OR "outcome* health" OR "risk trigger*" OR "trigger* risk*" OR "risk manifest*" OR "manifest* risk" OR risk-taking OR "protect* factor*" OR "exposure factor*" OR "risk* factor*" ) ) ) AND ( ( TITLE-ABS-KEY ( "Self-Help Groups" OR "Social Support" OR "risk factor modification" OR "risk factor management" OR "secondary prevention" OR "intervent* health* polic*" OR "health* intervention* polic*" OR "self-manag*" OR "self-efficac*" OR "self care" OR "self care" OR "social networking" OR "communit* engag*" OR "engag* communit*" OR "communit* participat*" OR "community participation" OR "cessation program*" OR "health* network*" OR "network* health*" OR "social* interact*" OR "interact* social*" OR "digital* rehab*" OR "digital* re-hab*" OR "re-hab* digital*" OR "rehab* digital*" OR "health promotion" OR "health education" OR ( "digital*" AND ( "health interven*" OR "interven* health" ) ) ) ) ) AND ( ( TITLE-ABS-KEY ( "rural health services" OR "rural population" OR "rural health" OR "rural hospitals" OR "rural nursing" OR "remot*" OR "rural*" ) ) ) | 22 |
| 8 | ( ( TITLE-ABS-KEY ( "Telemedicine" OR "Telerehabilitation" OR "Telehealth" OR "digital technolog*" OR "electronic technolog*" OR "mobile technolog*" OR "digital devic*" OR "electronic devic*" OR "mobile devic*" OR "monitor* devic*" OR "monitor* technolog*" OR "monitor* app*" OR e-health OR ehealth OR "electronic health" OR m-health OR mhealth OR "mobile health" OR "mobile app*" OR "digital app*" OR "electronic app*" OR "emerging technolog*" OR "emergent technolog*" OR "emerging digital technolog*" OR "emergent digital technolog*" OR "artificial* intelligen*" OR ai OR "machine learn*" OR ml OR "deep learn*" OR dl OR "blockchain" OR iot OR "internet of things" OR "immersive media" OR "augment* realit*" OR ar OR "virtual realit*" OR vr OR "mixed realit*" OR mr OR "digital screen*" OR "predict* analy*" OR robot* OR "fog comput*" OR nanotech* OR "cloud comput*" OR bionics OR "5G technolog*" OR "fifth generat* technolog*" OR "5G network*" OR "fifth generat* network*" OR "5G broadband" OR "5G cellular*" OR "fifth generat* cellular" OR "fifth generat* broadband" OR 3d OR 3-d OR "three dimens*" OR 3-dimens* OR cx OR "customer* experienc*" OR "Technology, High-Cost" OR "digital health" ) ) ) AND ( ( TITLE-ABS-KEY ( "Renal Arter* diseas*" OR "Renal Arter* Obstruct*" OR "Renal Arter* stenosis" OR "Renal Artery Obstruction" OR "kidney arter* diseas*" OR "myocardial infarction*" OR "heart attack*" OR "myocardial ischem*" OR "atrial fibrillation" OR afib OR atherosclerosis OR "peripheral arter* diseas*" OR stroke OR strokes OR "cerebral vascular accident*" OR "coronary arter* diseas*" OR "cardiovascular diseas*" ) ) ) AND ( ( TITLE-ABS-KEY ( "Attitud* to Health" OR "health* attitud*" OR "health* lifestyl*" OR "health* life styl*" OR "illness belie*" OR "behav* mod*" OR "mod* behav*" OR "behav* chang*" OR "Chang* behav*" OR "Health Behavi*" ) ) ) AND ( ( TITLE-ABS-KEY ( "behav* risk*" OR "risk* behav*" OR "exposure risk*" OR "risk* exposur*" OR "health hazard*" OR "hazard* health" OR "health outcome*" OR "outcome* health" OR "risk trigger*" OR "trigger* risk*" OR "risk manifest*" OR "manifest* risk" OR risk-taking OR "protect* factor*" OR "exposure factor*" OR "risk* factor*" ) ) ) AND ( ( TITLE-ABS-KEY ( "Self-Help Groups" OR "Social Support" OR "risk factor modification" OR "risk factor management" OR "secondary prevention" OR "intervent* health* polic*" OR "health* intervention* polic*" OR "self-manag*" OR "self-efficac*" OR "self care" OR "self care" OR "social networking" OR "communit* engag*" OR "engag* communit*" OR "communit* participat*" OR "community participation" OR "cessation program*" OR "health* network*" OR "network* health*" OR "social* interact*" OR "interact* social*" OR "digital* rehab*" OR "digital* re-hab*" OR "re-hab* digital*" OR "rehab* digital*" OR "health promotion" OR "health education" OR ( "digital*" AND ( "health interven*" OR "interven* health" ) ) ) ) ) AND ( LIMIT-TO ( LANGUAGE , "English" ) ) | 179 |
| 9 | ALL Languages | 243 |

|  | **EBSCO Host:**  **Psych Info, Health Source, Open Dissertation, Psych Article, Business Source Elite** |  |
| --- | --- | --- |
|  | **(13.05.2019 11.05)** |  |
| # | Search | Results |
| S1 | ( ( ( "Telemedicine"  OR  "Telerehabilitation"  OR  "Telehealth" ) )  OR  ( ( "digital technolog*"  OR  "electronic technolog*"  OR  "mobile technolog*" ) )  OR  ( ( "digital devic*"  OR  "electronic devic*"  OR  "mobile devic*" ) )  OR  ( ( "monitor* devic*"  OR  "monitor* technolog*"  OR  "monitor* app*" ) )  OR  ( ( e-health  OR  ehealth  OR  "electronic health" ) )  OR  ( ( m-health  OR  mhealth  OR  "mobile health" ) )  OR  ( ( "mobile app*"  OR  "digital app*"  OR  "electronic app*" ) )  OR  ( ( "emerging technolog*"  OR  "emergent technolog*"  OR  "emerging digital technolog*"  OR  "emergent digital technolog*" ) )  OR  ( ( "artificial* intelligen*"  OR  ai  OR  "machine learn*"  OR  ml  OR  "deep learn*"  OR  dl  OR  "blockchain"  OR  iot  OR  "internet of things" ) )  OR  ( ( "immersive media"  OR  "augment* realit*"  OR  ar  OR  "virtual realit*"  OR  vr  OR  "mixed realit*"  OR  mr  OR  "digital screen*" ) )  OR  ( ( "predict* analy*"  OR  robot*  OR  "fog comput*"  OR  nanotech*  OR  "cloud comput*"  OR  bionics ) )  OR  ( ( "5G technolog*"  OR  "fifth generat* technolog*"  OR  "5G network*"  OR  "fifth generat* network*"  OR  "5G broadband"  OR  "5G cellular*"  OR  "fifth generat* cellular"  OR  "fifth generat* broadband" ) )  OR  ( ( 3d  OR  3-d  OR  "three dimens*"  OR  3-dimens* ) )  OR  ( ( cx  OR  "customer* experienc*" ) )  OR  ( ( "Technology, High-Cost" ) )  OR  ( ( "digital health" ) ) ) | 709,901 |
| S2 | ( ( ( "Renal Arter* diseas*"  OR  "Renal Arter* Obstruct*"  OR  "Renal Arter* stenosis" ) )  OR  ( ( "Renal Artery Obstruction" ) )  OR  ( ( "kidney arter* diseas*" ) )  OR  ( ( "myocardial infarction*"  OR  "heart attack*"  OR  "myocardial ischem*" ) )  OR  ( ( "atrial fibrillation"  OR  afib ) )  OR  ( ( atherosclerosis ) )  OR  ( ( "peripheral arter* diseas*" ) )  OR  ( ( stroke  OR  strokes  OR  "cerebral vascular accident*" ) )  OR  ( ( "coronary arter* diseas*" ) )  OR  ( ( "cardiovascular diseas*" ) ) ) | 169,338 |
| S3 | ( ( ( "Attitud* to Health"  OR  "health* attitud*" ) )  OR  ( ( "health* lifestyl*"  OR  "health* life styl*" ) )  OR  ( ( "illness belie*" ) )  OR  ( ( "behav* mod*"  OR  "mod* behav*" ) )  OR  ( ( "behav* chang*"  OR  "Chang* behav*" ) )  OR  ( ( "Health Behavi*" ) ) ) | 219,392 |
| S4 | ( ( ( "behav* risk*"  OR  "risk* behav*" ) )  OR  ( ( "exposure risk*"  OR  "risk* exposur*" ) )  OR  ( ( "health hazard*"  OR  "hazard* health" ) )  OR  ( ( "health outcome*"  OR  "outcome* health" ) )  OR  ( ( "risk trigger*"  OR  "trigger* risk*" ) )  OR  ( ( "risk manifest*"  OR  "manifest* risk" ) )  OR  ( ( risk-taking ) )  OR  ( ( "protect* factor*" ) )  OR  ( ( "exposure factor*" ) )  OR  ( ( "risk* factor*" ) ) ) | 425,921 |
| S5 | ( ( ( "Self-Help Groups"  OR  "Social Support" ) )  OR  ( ( "risk factor modification"  OR  "risk factor management" ) )  OR  ( ( "secondary prevention" ) )  OR  ( ( "intervent* health* polic*"  OR  "health* intervention* polic*" ) )  OR  ( ( "self-manag*"  OR  "self-efficac*"  OR  "self care" ) )  OR  ( ( "self care"  OR  "social networking" ) )  OR  ( ( "communit* engag*"  OR  "engag* communit*"  OR  "communit* participat*" ) )  OR  ( ( "community participation" ) )  OR  ( ( "cessation program*" ) )  OR  ( ( "health* network*"  OR  "network* health*"  OR  "social* intereact*"  OR  "interact* social*" ) )  OR  ( ( "digital* rehab*"  OR  "digital* re-hab*"  OR  "re-hab* digital*"  OR  "rehab* digital*" ) )  OR  ( ( "health promotion"  OR  "health education" ) )  OR  ( ( ( "digital*" ) )  AND  ( ( "health interven*"  OR  "interven* health" ) ) )  OR  ( ( "health interven*"  OR  "interven* health" ) )  OR  ( ( "digital*" ) ) ) | 1,098,772 |
| S6 | "rural health services" OR "rural population" OR "rural health" OR "rural hospitals" OR "rural nursing" OR "remot*” or “rural*" | 356,184 |
| S7 | (S1 AND S2 AND S3 AND S4 AND S5 AND S6) | 9 |
| S8 | (S1 AND S2 AND S3 AND S4 AND S5) | 77 |

|  | **Embase**  **(08.05.2019 13.37)** |  |
| --- | --- | --- |
| # | Searches | Results |
| 1 | exp telemedicine/ | 34376 |
| 2 | exp telerehabilitation/ | 564 |
| 3 | 1 or 2 | 34376 |
| 4 | (digital technolog* or electronic technolog* or mobile technolog*).af. | 5303 |
| 5 | (digital devic* or electronic devic* or mobile devic*).af. | 12208 |
| 6 | (monitor* devic* or monitor* technolog* or monitor* app*).af. | 10145 |
| 7 | (e-health or ehealth or electronic health).af. | 30722 |
| 8 | (telehealth or tele health).af. | 8513 |
| 9 | (m-health or mhealth or mobile health).af. | 9021 |
| 10 | (telemedicine or tele-medicine).af. | 27858 |
| 11 | exp mobile application/ | 8369 |
| 12 | (mobile app* or digital app* or electronic app*).af. | 10078 |
| 13 | (emerging technolog* or emergent technolog* or emerging digital technolog* or emergent digital technolog*).af. | 7110 |
| 14 | (artificial* intelligen* or AI or machine learn* or ML or deep learn* or DL or blockchain or IoT or internet of things).af. | 1353438 |
| 15 | (immersive media or augment* realit* or AR or virtual realit* or VR or mixed realit* or MR or digital screen*).af. | 400237 |
| 16 | (predict* analy* or robot* or fog comput* or nanotech* or cloud comput* or bionics).af. | 168867 |
| 17 | (5G technolog* or fifth generat* technolog* or 5G network* or fifth generat* network* or 5G broadband or 5G cellular* or fifth generat* cellular or fifth generat* broadband).af. | 24 |
| 18 | (3D or 3-D or three dimens* or 3-dimens*).af. | 413395 |
| 19 | (CX or customer* experienc*).af. | 27333 |
| 20 | (technology high cost or technologies high cost or high cost technology or high cost technologies).af. | 69 |
| 21 | digital health.af. | 1560 |
| 22 | Exp Internet/ | 102933 |
| 22 | or/1-22 | 2370845 |
| 23 | exp cardiovascular disease/ | 3761315 |
| 24 | cardiovascular diseas*.af. | 1947383 |
| 25 | coronary arter* diseas*.af. | 223668 |
| 26 | (stroke or strokes or cerebral vascular accident*).af. | 459458 |
| 27 | peripheral arter* diseas*.af. | 20995 |
| 28 | atherosclerosis.af. | 245483 |
| 29 | (atrial fibrillation or Afib).af. | 126414 |
| 30 | (myocardial infarction* or heart attack* or myocardial ischem* or heart failure*).af. | 627778 |
| 31 | kidney arter* diseas*.af. | 5 |
| 32 | exp kidney artery stenosis/ | 11906 |
| 33 | (Renal Arter* diseas* or Renal Arter* Obstruct* or Renal Arter* stenosis).af. | 7769 |
| 34 | or/23-33 | 4300665 |
| 35 | exp health behavior/ | 379368 |
| 36 | (behav* chang* or Chang* behav*).af. | 59588 |
| 37 | (behav* mod* or mod* behav*).af. | 23830 |
| 38 | illness belie*.af. | 643 |
| 39 | (health* lifestyl* or health* life styl*).af. | 13819 |
| 40 | exp attitude to health/ | 104565 |
| 41 | (Attitud* to Health or health* attitud*).af. | 171510 |
| 42 | or/35-41 | 522407 |
| 43 | exp risk factor/ | 940681 |
| 44 | (risk* factor* and health).af. | 473025 |
| 45 | exposure factor*.af. | 1224 |
| 46 | protect* factor*.af. | 24259 |
| 47 | exp high risk behavior/ | 25239 |
| 48 | (risk manifest* or manifest* risk).af. | 219 |
| 49 | (risk trigger* or trigger* risk*).af. | 82 |
| 50 | (health outcome* or outcome* health).af. | 67228 |
| 51 | (health hazard* or hazard* health).af. | 57165 |
| 52 | (exposure risk* or risk* exposur*).af. | 4576 |
| 53 | (behav* risk* or risk* behav*).af. | 50070 |
| 54 | or/43-53 | 1214576 |
| 55 | (digital* and (health interven* or interven* health)).af. | 299 |
| 56 | exp health promotion/ or exp health education/ | 302333 |
| 57 | (digital* rehab* or digital* re-hab*or re-hab* digital* or rehab* digital*).af. | 16 |
| 58 | (health* network* or network* health* or (social* intereact* or interact* social*)).af. | 45038 |
| 59 | cessation program*.af. | 6039 |
| 60 | exp community participation/ | 1580 |
| 61 | (communit* engag* or engag* communit* or communit* participat*).af. | 9155 |
| 62 | exp self care/ | 74580 |
| 63 | exp social network/ | 14047 |
| 64 | (self-manag* or self-efficac* or self care).af. | 93169 |
| 65 | (intervent* health* polic* or health* intervention* polic*).af. | 36 |
| 66 | secondary prevention.af. | 38613 |
| 67 | (risk factor modification or risk factor management).af. | 3119 |
| 68 | exp self help/ | 12993 |
| 69 | exp social support/ | 83850 |
| 70 | or/55-69 | 567887 |
| 71 | exp rural health care/ | 12974 |
| 72 | exp rural health/ | 710 |
| 73 | exp rural population/ | 41642 |
| 74 | (rural hospital* or hospital* rural).af. | 4789 |
| 75 | exp rural health nursing/ | 143 |
| 76 | (rural* or remot*).af. | 283312 |
| 77 | or/71-76 | 283312 |
| 78 | 22 and 34 and 42 and 54 and 70 and 77 | 27 |
| 79 | 22 and 34 and 42 and 54 and 70 | 460 |
| 80 | 78 or 79 | 460 |
| 81 | limit 80 to english language | 438 |

|  | **GREYLIT.ORG**  **(14.05.2019 11.19)** |  |
| --- | --- | --- |
| # | Searches | Results |
| 1 | Cardiovascular | 115 |
| 2 | Digital | 7510 |
| 3 | Digital AND Cardiovascular | 116 |

**Table 4: Table of included studies**

| **No.** | **Author** | **Title** | **Journal** | **Year** |
| --- | --- | --- | --- | --- |
| 1 | Frederix I, Driessche NV, Hansen D, et al. | Increasing the medium-term clinical benefits of hospital-based cardiac rehabilitation by physical activity telemonitoring in coronary artery disease patients. | Eur J Prev Cardiol. 2015:22(2): 150-158. | 2015 |
| 2 | Reid RD, Morrin LI, Beaton LJ, et al. | Randomized trial of an Internet-based computer-tailored expert system for physical activity in patients with heart disease. | Eur J Prev Cardiol. 2012; 19(6): 1357-1364. | 2012 |
| 3 | Southard BH, Southard DR, Nuckolls J. | Clinical trial of an Internet-based case management system for secondary prevention of heart disease. | J Cardiopulm Rehabil. 2003;23(5):341-348 | 2003 |
| 4 | Vemooij JW, Kaasjager HA, van der Graaf Y, et al | SMART Study Group. Internet based vascular risk factor management for patients with clinically manifest vascular disease: randomised controlled trial. | BMJ. 2012;344:e3750. | 2012 |
| 5 | Chow CK, Redfern J, Hillis GS, et al. | Effect of lifestyle-focused text messaging on risk factor modification in patients with coronary heart disease. | JAMA. 2015;314:1255-1263. | 2015 |
| 6 | Kamal AK, Shaikh Q, Pasha O, et al. | A randomized controlled behavioral intervention trial to improve medication adherence in adult stroke patients with prescription tailored short messaging service (SMS)-SMS4Stroke study. | BMC Neurol. 2015;15:212. | 2015 |
| 7 | Khonsari S, Subramanian P, Chinna K, et al. | Effect of a reminder system using an automated short message service on medication adherence following acute coronary syndrome. | Eur J Cardiovasc Nurs. 2014;14:170-179. | 2014 |
| 8 | Maddison R, Pfaeffli L, Whittaker R, et al. | A mobile phone intervention increases physical activity in people with cardiovascular disease: Results from the HEART randomized controlled trial. | Eur J Prev Cardiol. 2015;22:701-709. | 2015 |
| 9 | Park LG, Howie-Esquivel J, Dracup K. | A text messaging intervention to promote medication adherence for patients with coronary heart disease: a randomized controlled trial. | J Adv Nurs. 2014;70:1932-1953. | 2014 |
| 10 | Pfaeffli Dale L, Whittaker R, Jiang Y, et al. | Text message and internet support for coronary heart disease self-management: results from the Text4Heart randomized controlled trial. | J Med Internet Res. 2015;17:e237. | 2015 |
| 11 | Quilici J, Fugon L, Beguin S, et al. | Effect of motivational mobile phone short message service on aspirin adherence after coronary stenting for acute coronary syndrome. | Int J Cardiol. 2013;168:568-569. | 2013 |
| 12 | Devi R, Powell J, Singh S. | A web-based program improves physical activity outcomes in a primary care angina population: randomized controlled trial. | Journal of Medical Internet Research 2014;16(9):e186. | 2014 |
| 13 | Lear SA, Singer J, Banner-Lukaris D et al. | Randomized trial of a virtual cardiac rehabilitation program delivered at a distance via the internet. | Circulation: Cardiovascular Quality and Outcomes 2014;7(6):952–9 | 2014 |
| 14 | Dale LP, Whittaker R, Jiang Y et al | Text message and internet support for coronary heart disease self-management: results from the Text4Heart randomized controlled trial. | Journal of Medical Internet Research 2015;17(10):e237. | 2015 |
| 15 | Pandey AK, Choudhry N. | Text message reminders to address medication non-adherence in post-MI patients: A one year intervention study. | Canadian Journal of Cardiology 2014;1:S179. | 2014 |
| 16 | Widmer RJ, Allison TG, Lennon R, et al. | Digital health intervention during cardiac rehabilitation: A randomized controlled trial. | American Heart Journal Volume 188, June 2017, Pages 65-72 | 2017 |
| 17 | Johnston N, Bodegard J, Jerström S, et al | Effects of interactive patient smartphone support app on drug adherence and lifestyle changes in myocardial infarction patients: A randomized study. | Am Heart J. 2016 Aug;178:85-94. | 2016 |
| 18 | Hawkes AL, Patrao TA, Atherton J et al | Effect of a telephone-delivered coronary heart disease secondary prevention program (proactive heart) on quality of life and health behaviours: primary outcomes of a randomised controlled trial. | nt J Behav Med. 2013 Sep;20(3):413-24 | 2013 |
| 19 | Wan LH, Zhang XP, Mo MM, Xiong XN et al | Effectiveness of Goal-Setting Telephone Follow-Up on Health Behaviors of Patients with Ischemic Stroke: A Randomized Controlled Trial. | J Stroke Cerebrovasc Dis. 2016 Sep;25(9):2259-70. | 2016 |
| 20 | Jos J Kraal, Niels Peek et al. | Effects of home-based training with telemonitoring guidance in low to moderate risk patients entering cardiac rehabilitation: short-term results of the FIT@Home study | European Journal of Preventive Cardiology 2014, Vol. 21(2S) 26–31 | 2014 |
| 21 | Laila M. Akhu-Zaheya∗, Wa’ed Y. Shiyab | The effect of short message system (SMS) reminder on adherence to ahealthy diet, medication, and cessation of smoking among adultpatients with cardiovascular diseases | International Journal of Medical Informatics 98 (2017) 65–75 | 2017 |
| 22 | Joachim Ögren, Anna-Lotta Irewall, Lars Söderström et al | Long-term, telephone-based follow-up after stroke and TIA improves risk factors: 36-month results from the controlled NAILED stroke risk factor trial randomized | BMC Neurology (2018) 18:153 | 2018 |
| 23 | Margarite J. Vale, Michael V. Jelineka,, et al. | Coaching patients with coronary heart disease to achieve the target cholesterol: A method to bridge the gap “real world”—randomized controlled trialbetween evidence-based medicine and the | Journal of Clinical Epidemiology 55 (2002) 245–252 | 2002 |
| 24 | J Redfern, T Briffa, E Ellis, S B Freedman | Choice of secondary prevention improves risk factors after acute coronary syndrome: 1-year follow-up of the CHOICE (Choice of Health Options In prevention of Cardiovascular Events) randomised controlled trial | Heart 2009;95:468-475. | 2009 |
| 25 | Tiede M, Dwinger S, Herbarth L et al. | Long-term effectiveness of telephone-based health coaching for heart failure patients: A post-only randomised controlled trial | Journal of Telemedicine and Telecare (2017) 23(8) 716-724 | 2017 |

**Table 5: Table of excluded studies**

| **No.** | **Publication No.** | **Title** | **Reason for exclusion** |
| --- | --- | --- | --- |
| 1 | Am J Cardiol 2002; 89: 1263–1268. | Effectiveness of three models for comprehensive cardiovascular disease risk reduction. | A Model |
| 2 | Monaldi Arch Chest Dis 2006; 66: 8–12. | Efficacy of telecardiology in improving the results of cardiac rehabilitation after acute myocardial infarction. | A Program |
| 3 | BMJ Open. 2012;2:e000606. doi: 10.1136/bmjopen-2011-000606. | Design and rationale of the tobacco, exercise and diet messages (TEXT ME) trial of a text message-based intervention for ongoing prevention of cardiovascular disease in people with coronary disease: a randomised controlled trial protocol. | A Protocol |
| 4 | BMC Cardiovascular Disorders 2013, 13:82 | Effects and costs of home-based training with telemonitoring guidance in low to moderate risk patients entering cardiac rehabilitation: The FIT@Home study | A Protocol |
| 5 | BMJ Open 2019;9:e024269. | Design and rationale of the MyHeartMate study: a randomised controlled trial of a game-based app to promote behaviour change in patients with cardiovascular disease | A Protocol |
| 6 | Circ Cardiovasc Qual Outcomes. 2011 Mar;4(2):235-42. | Secondary prevention risk interventions via telemedicine and tailored patient education (SPRITE): a randomized trial to improve postmyocardial infarction management. | A Protocol |
| 7 | BMC Cardiovasc Disord. 2017 Jan 31;17(1):46 | Effects of cardiac telerehabilitation in patients with coronary artery disease using a personalised patient-centred web application: protocol for the SmartCare-CAD randomised controlled trial. | A Protocol |
| 8 | BMJ Open Sport Exerc Med. 2019 Jul 5;5(1):e000539 | Personalised eHealth intervention to increase physical activity and reduce sedentary behaviour in rehabilitation after cardiac operations: study protocol for the PACO randomised controlled trial (NCT03470246) | A Protocol |
| 9 | J Clin Nurs. 2018 Dec;27(23-24):4311-4320 | The effect of mobile messaging apps on cardiac patient knowledge of coronary artery disease risk factors and adherence to a healthy lifestyle. | A Quasi-Experiment |
| 10 | J Clin Neurol. 2015;11:142-148. | Feasibility of using a mobile application for the monitoring and management of stroke-associated risk factors. | Feasibility Cross Sectional Study |
| 11 | Journal of Clinical Nursing 2016; 25(5-6):664–71. | Electronic messaging support service programs improve adherence to lipid-lowering therapy among outpatients with coronary artery disease: an exploratory randomised control study. | Intra DHI Program |
| 12 | J Neurol Sci. 2015 Nov 15;358(1-2):535-7. | Mobile health medication adherence and blood pressure control in recent stroke patients. | Letter to the Editor |
| 13 | J Med Internet Res. 2014;16:e77. | An Internet- and mobile-based tailored intervention to enhance maintenance of physical activity after cardiac rehabilitation: Short-term results of a randomized controlled trial. | Low participation study |
| 14 | JMIR Mhealth Uhealth. 2019 Apr; 7(4): e10874. | Evaluating the Impact of the HeartHab App on Motivation, Physical Activity, Quality of Life, and Risk Factors of Coronary Artery Disease Patients: Multidisciplinary Crossover Study | Low participation study |
| 15 | CMAJ. 2007; 177(8):859-865. | One-year follow-up of a therapeutic lifestyle intervention targeting cardiovascular disease risk. | Mixed Diagnosis Population |
| 16 | Clinical Rehabilitation. 31(8):1068–1077, AUGUST 2017 | Home exercise programmes supported by video and automated reminders compared with standard paper-based home exercise programmes in patients with stroke: a randomized controlled trial | No Clinical Care Control |
| 17 | Eur J Heart Fail. 2012; 14(3)333-340. | Effect of a telemonftoring-facilitated collaboration between general practitioner and heart failure clinic on mortality and rehospitalization rates in severe heart failure : the TEMA-HF I (TeIemonitoring in the MAnagement of Heart Failure) study. | No Clinical Risk Factor |
| 18 | J Med Internet Res. 2009;11(3):e34. | Mobitel Investigators. Effect of home-based telemonitoring using mobile phone technology on the outcome of heart failure patients after an episode of acute decompensation: randomized controlled trial. | No Clinical Risk Factor |
| 19 | Circulation. 2011;123:1873-1880. | Impact of remote telemedical management on mortality and hospitalizations in ambulatory patients with chronic heart failure: The telemedical interventional monitoring in heart failure study. | No Clinical Risk Factor |
| 20 | J Med Internet Res. 2009;11:e34. | Effect of home-based telemonitoring using mobile phone technology on the outcome of heart failure patients after an episode of acute decompensation: Randomized controlled trial. | No Clinical Risk Factor |
| 21 | Heart. 2014;100:1770-1779. | Smartphone-based home care model improved use of cardiac rehabilitation in postmyocardial infarction patients: Results from a randomised controlled trial. | No Clinical Risk Factor |
| 22 | J Med Internet Res. 2014;16:e282. | Use of home telemonitoring to support multidisciplinary care of heart failure patients in Finland: Randomized controlled trial. | No Clinical Risk Factor |
| 23 | J Cardiovasc Transl Res. 2015;8:283-292. | Digital health intervention as an adjunct to cardiac rehabilitation reduces cardiovascular risk factors and rehospitalizations. | No Clinical Risk Factor |
| 24 | BMC Med Res Methodol 2005; 5: 18. | Recruitment of ethnic minority patients to a cardiac rehabilitation trial: The Birmingham Rehabilitation Uptake Maximisation (BRUM) study [ISRCTN72884263]. | No Digital Health Intervention |
| 25 | Age Ageing 2011; 40: 78–85. | Home-based cardiac rehabilitation is as effective as center-based cardiac rehabilitation among elderly with coronary heart disease: Results from a randomised clinical trial. | No Digital Health Intervention |
| 26 | Am J Phys Med Rehabil 2006; 85: 711–717. | Cardiac rehabilitation versus home exercise after coronary artery bypass graft surgery: A comparison of heart rate recovery. | No Digital Health Intervention |
| 27 | Medicine (2018) 97:35(e12069) | Home-based telehealth exercise training program in Chinese patients with heart failure: A randomized controlled trial | Non behavioural or clinical Risk Factor |
| 28 | J Med Internet Res. 2014;14:e31. | Mobile phone-based telemonitoring for heart failure management: A randomized controlled trial. | Non behavioural or clinical Risk Factor |
| 29 | J Med Internet Res. 2012 Feb 16;14(1):e31. | Mobile phone-based telemonitoring for heart failure management: a randomized controlled trial. | Non behavioural or clinical Risk Factor |
| 30 | J Telemed Telecare. 2009;15(1):46-50 | Outcomes of a home telehealth intervention for patients with heart failure. | Non behavioural or clinical Risk Factor |
| 31 | Health Education Research 2009;24(4):646–54. | The health impact of an online heart disease support group: a comparison of moderated versus unmoderated support. | Non standardized Measurement Unit |
| 32 | J Med Internet Res. 2014 Dec 11;16(12):e282. | Use of home telemonitoring to support multidisciplinary care of heart failure patients in Finland: randomized controlled trial. | Not published |
| 33 | BMC Cardiovascular Disorders 2012, 12:47 | The effects of Hartcoach, a life style intervention provided by telephone on the reduction of coronary risk factors: a randomised trial. | Ongoing |
| 34 | Circulation 1984; 70: 645–649. | Home versus group exercise training for increasing functional capacity after myocardial infarction. | Out-dated |
| 35 | Ann Intern Med. 1994;120(9):721-729. | A Case-Management System for Coronary Risk Factor Modification after Acute Myocardial Infarction | Out-dated |
| 36 | Telemed J E Health. 2007; 13(3):323-330. | Utilization of the Internet to deliver cardiac rehabilitation at a distance: a pilot study | Pilot Study |
| 37 | Telemed J E-Health . 2007 ; 13 : 323-330 . | Utilization of the Internet to deliver cardiac rehabilitation at a distance: a pilot study | Pilot Study |
| 38 | Educ Couns . 2008 ; 73 : 67-72 . | A pilot-study to identify the feasibility of an Internet-based coaching programme for changing the vascular risk profile of high-risk patients. | Pilot Study |
| 39 | J Med Internet Res. 2013;15:e53. | A text messaging intervention to improve heart failure self-management after hospital discharge in a largely African–American population: Before-after study. | Pilot Study |
| 40 | PLoS One. 2011;6:e14669. | Development and feasibility of a smartphone, ECG and GPS based system for remotely monitoring exercise in cardiac rehabilitation. | Pilot Study |
| 41 | Patient Educ Couns. 2007;66:319–326. doi: 10.1016/j.pec.2007.01.005. | Interactive voice response telephony to promote smoking cessation in patients with heart disease: a pilot study. | Pilot Study |
| 42 | J Med Internet Res. 2018 Nov 19;20(11):e12052. | Evaluation of a Web-Based Intervention for Multiple Health Behavior Changes in Patients with Coronary Heart Disease in Home-Based Rehabilitation: Pilot Randomized Controlled Trial. | Pilot study |
| 43 | Telemed J E Health. 2007 Jun;13(3):323-30. | Utilization of the internet to deliver cardiac rehabilitation at a distance: a pilot study. | Pilot Study |
| 44 | Top Stroke Rehabil. 2016 Jun;23(3):170-7 | Increasing physical activity in stroke survivors using STARFISH, an interactive mobile phone application: a pilot study. | Pilot Study, Low participation study |
| 45 | Journal of the American Society of Hypertension Volume 10, Issue 4, Supplement, April 2016, Page e65 | Effect on blood pressure of integrating a smartphone-based self-management system into the care of patients with advanced chronic kidney disease | RCT Rationale Test Cross-Sectional |
| 46 | Eur J Prev Cardiol, [in press]. http://dx.doi.org/10.1177/2047487315613460 | The effectiveness of mobile-health behaviour change interventions for cardiovascular disease self-management: a systematic review. | Systematic Literature Review |
| 47 | Cochrane Database of Systematic Reviews 2017, Issue 4. Art. No.: CD011851. | Mobile phone textmessaging to improve medication adherence in secondary prevention of cardiovascular disease. | Systematic Literature Review |
| 48 | Cochrane Database of Systematic Reviews 2015, Issue 12. Art. No.: CD009386. | Internet-based interventions for the secondary prevention of coronary heart disease. | Systematic Literature Review |
| 49 | European Journal of Preventive Cardiology 2015, Vol. 22(8) 959–971 | Telehealth interventions versus center-based cardiac rehabilitation of coronary artery disease: A systematic review and meta-analysis | Systematic Literature Review |
| 50 | http://dx.doi.org/10.1016/j.pcad.2016.03.002 0033-0620/© 2016 Elsevier Inc. All rights reserved. | Mobile Phone Interventions for the Secondary Prevention of Cardiovascular Disease | Systematic Literature Review |
| 51 | 2014 Wolters Kluwer Health | Lippincott Williams & Wilkins. | Primary and Secondary Prevention of Cardiovascular Disease | Systematic Literature Review |
| 52 | 2015 American Heart Association, Inc. Circulation is available at http://circ.ahajournals.org | Mobile Health Devices as Tools for Worldwide Cardiovascular Risk Reduction and Disease Management | Systematic Literature Review |
| 53 | j.mayocp.2014.12.026 | Digital health interventions for the prevention of cardiovascular disease: a systematic review and metaanalysis | Systematic Literature Review |
| 54 | European Journal of Cardiovascular Prevention and Rehabilitation 2009, 16:281–289 | Telehealth interventions for the secondary prevention of coronary heart disease: a systematic review | Systematic Literature Review |
| 55 | Heart & Lung 47 (2018) 351–359 | Mobile phone text messaging for improving secondary prevention in cardiovascular diseases: A systematic review | Systmatic LitRvw |
| 56 | PhD Thesis, University of London, 1998. | A comparison of a multi-disciplinary home based cardiac rehabilitation program with comprehensive conventional rehabilitation in post-myocardial infarction patients. | Thesis |
| 57 | BMJ Innovations Published Online First: 14 March 2019 | Development of an online secondary prevention programme for stroke survivors: Prevent 2nd Stroke | Usability/Acceptability Test study |

Table of excluded studies

1. **Abbreviations**

RCT - Randomized Controlled Trial
RCT - Randomized Clinical Trial
CHD - Coronary Heart Disease
CABG - coronary artery bypass graft surgery
PCI - percutaneous coronary intervention
PCR - Percutaneous coronary revascularization
ACS - Acute Coronary Syndrome
TC - Total Cholesterol
TG - Triglycerides
HDL - High-density Lipoprotein
LDL - Low-density Lipoprotein
CHF - Congestive Heart Failure
ST - Stent Thrombosis
MI - Myocardial infarction
CRP - Cardiac Rehabilitation Programs
vCRP - Virtual Cardiac Rehabilitation Programs
CVD - Cardiovascular Diseases
ISH - Ischaemic Heart Disease
IS - Ischemic Stroke
HF - Heart Failure
TIA - Transient Ischemic Attack
CAD - Coronary Artery Disease

SMS - Short Message Services

BMI - Body Mass Index

TC - Total Cholesterol

HDL - High Density Lipoprotein

LDL - Low Density Lipoprotein

TG - Triglycerides

SBP - Systolic Blood Pressure

DBP - Diastolic Blood Pressure

PA - Physical Activity

PI - Physical Inactivity

HbA1c - Glycated haemoglobin (blood sugar)

MMAS 8 - Morisky Medication Adherence Scale 8

MH - Mantel-Haenszel

IV - Inverse-Variance

1. **Definition of terms**

**Digital health technologies:** the internet, phone applications and devices, and monitoring sensors which are used for telemedicine, web-browsing, e-mailing, text messaging, and monitoring in the secondary prevention and management of chronic health conditions.

**Mobile phones:** cell phones and smartphones capable of using subscriber identification module, SIM cards.

**Cell phones:** communication device without advanced digital operating features

**Smart phones:** combination of digital device applications in one unit with advances digital operating features e.g. touch screen, cookies etc.

**Wearables:** electronic devices worn on the body with ability to sense and communicate physiological changes from the body of the user and user’s environment to a central digital base.

**Personal computers:** a small computer with a microprocessor, designed for use by an individual. An example of personal computers are desktop computers used in homes, schools, and small businesses.

**Telerehabilitation:** the delivery of cardiac rehabilitation services over telecommunication networks and the internet. Telerehabilitation allows patients to interact with providers remotely and can be used both to assess patients and to deliver therapy.

**Telemonitoring:** the use of information and communication technologies to record and transmit patient’s health behaviour change activity data between the patient and the health centre.

**Online support:** health behaviour change support given over the internet

**Online coaching:** Health behaviour change coaching delivered over the internet.
