# Supplementary material for: Digital Technology Interventions for Risk Factor Modification in Patients With Cardiovascular Disease: Systematic Review and Meta-analysis
Source: JMIR Mhealth Uhealth. 2021 Mar 3;9(3):e21061. doi: 10.2196/21061 (PMC7970167; doi:10.2196/21061)
Supplement: Multimedia Appendix 2 [file mhealth_v9i3e21061_app2.doc]

**Multimedia Appendix 2.** Study characteristics.

**Table 6**

|  | **Research** | **Method** | **Participants** | | | | **Interventions** | | **Outcomes** | **Population** | | | **Digital Device** | | | **Change Techniques** | |
| --- | --- | --- | --- | --- | --- | --- | --- | --- | --- | --- | --- | --- | --- | --- | --- | --- | --- |
| **No.** | **Author, Year** | **RCT Pattern** | **n (% Male)** | **Mean Age** | **Region** | **Digital Strategy** | | **Follow-Up Period** | **Behavioural & Clinical** | | **Diagnosis ^a^** | **Device** | | **Brand** | **Behavioural Constructs** | |  |
| 1 | Vale et al, 2002 | (A Methodical Model System), DI/UC | 219 (75%) | 61.5 | Australia | Telephone Coaching | | (6 months post randomization) | TC, HDL, LDL and TG | | CHD (who had CABG or PCR hospitalization) | Cell Phone | | (COACH) | Support, Self-Management | |  |
| **Note on Author's Study Summary**: Availability of professional coach with evidence-based advice for risk factor modification. | | | | | | | | | | | | | | | | | |
| 2 | Southard et al, 2003 | (Home-based Internet Program) DI/UC | 104 (75%) | 62.3 | USA | Online feedback and tracking | | (6 months) | BMI, SBP, DBP, TC, HDL, LDL, TG, Physical Activity, Smoking cessation and Healthy food intake | | General CVD: CHD, CHF. | Smartphone Application | | HeartLinks | Feedback mechanism, Progress Monitoring and Incentives (souvenirs) | |  |
| **Note on Author's Study Summary**: An alternative for patients unwilling to partake in hospital rehabilitation programs or an adjunct to cardiac rehabilitation | | | | | | | | | | | | | | | | | |
| 3 | Redfern et al, 2009 | (Single-blinded Study with core module), DI+UC/UC | 144 (74%) | 64.5 | Australia | SMS Telephone Support | | (12 months) | BMI, TC, HDL, LDL, TG, SBP, DBP, Physical Activity, Physical Inactivity (sedentary), Smoking | | ACS survivors (not accessing standard cardiac rehabilitation) | Cell Phone | | (CHOICE) | Persuasion, Self-Management, Customization | |  |
| **Note on Author's Study Summary**: Programme significantly improved the modifiable risk profiles and risk factor knowledge | | | | | | | | | | | | | | | | | |
| 4 | Reid et al, 2012 | (A Health Application), DI/UC | 223 (84%) | 56.4 | Canada | Telerehabilitation | | (6 months) | Physical Activity | | ACS (underwent successful PCR) | Internet website | | (CardioFit) | Telemonitoring, Tracking, Self- Management, Customization | |  |
| **Note on Author's Study Summary**: Study efficiently bridged the gap on cost, time, distance, mobility and willing to participate in cardiac rehabilitation programs. | | | | | | | | | | | | | | | | | |
| 5 | Vernooij et al, 2012 | (Multicentre, Prospective Study), DI+UC/UC | 314 (75%) | 59.95 | Netherlands | Telemonitoring | | (12 months) | BMI, SBP, DBP, TC, HDL, LDL, TG, Glucose, HbA1_C_, Smoking | | Atherosclerosis | Online Web | |  | Self-manage- ment, Cognition (Progress data recording and tracking) and Support | |  |
| **Note on Author's Study Summary**: Study clinical importance effect is small and limited in all variables except in LDL and smoking. | | | | | | | | | | | | | | | | | |
| 6 | Hawkes et al, 2012 | (A Parallel Prospective Study, Prevention Program), DI/UC | 430 (75%) | 60.6 | Australia | Telerehabilitation | | (6 months) | BMI, Physical Activity, Alcohol Intake, Smoking | | CHD | Cell Phone | | (ProActive Heart) | Self-efficacy, Self-management, Social Supports, Perceived Risks and Perceived Benefits | |  |
| **Note on Author's Study Summary**: Study evidenced optimum participation and effective remote access option | | | | | | | | | | | | | | | | | |
| 7 | Quilici et al, 2012 | (A Pilot study), DI/UC | 499 (77%) | 64 | France | Telemedicine (SMS Telephone Support) | | (1 month) | Medication adherence | | ACS (ST Prevention) | Cell Phone | |  | Cognition, Support, Customization | |  |
| **Note on Author's Study Summary**: Early first month adherence after ACS hospital discharge could be cost-effective to improve patient outcome after ACS. | | | | | | | | | | | | | | | | | |
| 8 | Park et al, 2013 | DI+EDU/ EDU/UC | 90 (76%) | 59.2 | USA | Telemedicine (SMS Telephone support) | | (1 month) | Medication adherence | | MI, PCI, MI and PCI (CHD) | Cell Phone | | (MEMS) | Cognition, Monitoring, Customization, Gift reward and reimbursement | |  |
| **Note on Author's Study Summary**: Early first month adherence (using mobile phones) following MI and/or PCI hospital discharge reduces morbidity and mortality | | | | | | | | | | | | | | | | | |
| 9 | Devi et al, 2014 | DI/UC | 94 (74%) | 66.24 | United Kingdom | Telerehabilitation | | (1.5 months, 6 months) | Weight, SBP, DBP, Physical Activity, Physical Inactivity (sedentary) | | Angina (CHD) | Internet Web | | (ActivateYourHeart) | Support, Self-management | |  |
| **Note on Author's Study Summary**: Internet-based approach may offer an alternative self-management approach | | | | | | | | | | | | | | | | | |
| 10 | Lear et al, 2014 | (CRP), DI/UC | 78 (85%) | 60.1 | Canada | Telerehabilitation | | (4, 12, and 16 months) | BMI, TC, HDL, LDL, SBP, DBP, TG, Glucose, Physical Activity, Smoking, Healthy food intake | | General CVD | Internet Web | | (vCRP) | Self-management, Perceived risks and perceived benefits, Support | |  |
| **Note on Author's Study Summary**: Virtual CRP is a safe and effective at providing sustainable improvements in exercise capacity and reductions in CVD risk. | | | | | | | | | | | | | | | | | |
| 11 | Maddison et al, 2014 | (Single-blinded, Parallel, Two-arm Study), DI +UC/UC | 171 (81%) | 60.2 | New Zealand | Telerehabilitation | | (6 months) | Physical Activity | | Angina, MI, CHD (IHD) | Smartphone Application | | (HEART) | Cognition, Self-efficacy, Social support, Motivation, Customization | |  |
| **Note on Author's Study Summary**: A mobile phone intervention was not effective at increasing exercise capacity over and above usual care | | | | | | | | | | | | | | | | | |
| 12 | Pandey et al, 2014 | DI/UC | 100 (59%) | 63 | Canada | Telemedicine (SMS Telephone Support) | | (12 months) | Medication adherence | | MI | Cell phone | |  | Cognition (auro-reminder) | |  |
| **Note on Author's Study Summary**: A simple and scalable method for improving adherence to evidence-based therapies - health outcome impact not yet established | | | | | | | | | | | | | | | | | |
| 13 | Kraal et al, 2014 | DI/UC | 200 (68%) | 135 | Netherlands | Telemonitoring | | (3 months) | Physical Activity | | General CVD: MI, Unstable Angina, PCI or CABG (CHD) | Internet web | | (FIT@Home) | Motivational interviewing, Monitoring and feedback, Self-Management | |  |
| **Note on Author's Study Summary**: Home-based telemonitoring is an effective alternative for regular centre-based usual care | | | | | | | | | | | | | | | | | |
| 14 | Kamal et al, 2015 | (parallel, assessor-blinded, superiority study), DI +UC/UC | 200 (68%) | 56.8 | Pakistan | Telemedicine (SMS Telephone Support) | | (2 months) | DBP, Medication adherence | | Stroke survivors | Cell Phone | | (SMS4Stroke) | Cognition, Customization, Social support | |  |
| **Note on Author's Study Summary**: Authors feel that the SMS intervention seems feasible for clinical use in stroke survivors for improving adherence | | | | | | | | | | | | | | | | | |
| 15 | Khonsari et al, 2015 | DI/UC | 62 (85%) | 57.9 | Malaysia | Telemedicine (SMS Telephone Support) | | (2 months) | Medication adherence | | ACS | Cell Phone | |  | Cognition | |  |
| **Note on Author's Study Summary**: Study concludes an automated SMS-based reminder system can potentially enhance medication adherence in ACS patients during the early post-discharge period | | | | | | | | | | | | | | | | | |
| 16 | Chow et al, 2015 | DI+UC/UC | 710 (82%) | 58 | Australia | Telerehabilitation (SMS Telephone support) | | (6 months) | BMI, TC, HDL, LDL, SBP, DBP, TG, Physical Activity, Physical Inactivity (Sedentary), Smoking cessation, Medication adherence | | CHD | Cell Phone | |  | Motivation, Cognition, Persuasion, Customization, Support | |  |
| **Note on Author's Study Summary**: Use of a lifestyle-focused text messaging service compared with usual care resulted in a modest improvement in LDL-C level and greater improvement in other cardiovascular disease risk factors | | | | | | | | | | | | | | | | | |
| 17 | Frederix et al, 2013 | DI/UC | 80 (83%) | 60.5 | Belgium | Telemonitoring | | (6 months) | TC, HDL, LDL, TG, SBP, DBP, Glucose, HbA1_C_ Physical Activity, | | CAD | Motion Sensor | |  | Perceived benefit, Self- management, Monitoring and feedback | |  |
| **Note on Author's Study Summary**: Telemonitoring program might be an effective intervention to maintain exercise tolerance | | | | | | | | | | | | | | | | | |
| 18 | Dale et al, 2015 | (Two-arm, Parallel study) DI+UC/UC | 123 (81%) | 59.5 | New Zealand | SMS Telephone support | | (3, 6 months) | BMI, TC, HDL, LDL, SBP, DBP, Physical Activity, Healthy food intake, Medication adherence, Smoking, Alcohol intake | | CHD | Cell Phone | | (Text4  Heart) | Cognition, Support, Self-management | |  |
| **Note on Author's Study Summary**: Intervention plus usual care showed a positive effect on adherence to multiple lifestyle behaviour changes at 3 months | | | | | | | | | | | | | | | | | |
| 19 | Johnston et al, 2016 | (A multicentre Study), DI+UC/UC | 166 (81%) | 57.6 | Sweden | Telemonitoring | | (6 months) | BMI, LDL, SBP, Physical Activity, Smoking | | MI | Smartphone Application | | (SUPPORT) | Feedback, Customization, Motivation, Support | |  |
| **Note on Author's Study Summary**: Intervention tool improved patient self-reported medication adherence and may be associated with trend in improved lifestyle changes and Quality of Life. | | | | | | | | | | | | | | | | | |
| 20 | Wan et al, 2016 | (Multicentre, Assessor blinded, Parallel Study), DI/UC | 80 (71%) | 59.66 | China | Telerehabilitation | | (6 months) | Physical Activity, Medication adherence, Alcohol intake | | IS | Cell Phone | |  | Self-management, Cognition | |  |
| **Note on Author's Study Summary**: None | | | | | | | | | | | | | | | | | |
| 21 | Akhu-Z et al, 2016 | DI/Placebo/UC | 180 (54%) | 54.9 | Jordan | Telemedicine (SMS Telephone support) | | (3 months) | Medication adherence, Healthy food intake | | General CVD | Cell Phone | |  | Self- management (Self- Regulation Theory) | |  |
| **Note on Author's Study Summary**: Study documents that SMS is effective in improving adherence in health outcomes | | | | | | | | | | | | | | | | | |
| 22 | Tiede et al, 2017 | DI/UC | 136 (50%) | 74 | Germany | Telephone Coaching | | (24, 48 months) | Physical Inactivity (Sedentary), Unhealthy food intake, Smoking, Alcohol intake | | HF | Cell Phone | | (TBHC) | Perceived benefit, Self- management | |  |
| **Note on Author's Study Summary**: Study results suggest that telephone-based health coaching has no effect on QoL, anxiety and depression of heart failure patients, but helps in improving certain risk behaviours and changes the locus of control to be more externalised | | | | | | | | | | | | | | | | | |
| 23 | Widmer et al, 2017 | (Adjunct study), DI+UC/UC | 44 (82%) | 65.3 | USA | Telerehabilitation | | (3 months Post randomization) | Weight, BMI, TC, HDL, LDL, TG, SBP, DBP, Glucose, Physical Activity, Medication adherence, Healthy food intake, Smoking | | MI | Smartphone Application | | (PHA) | Support and Feedback | |  |
| **Note on Author's Study Summary**: Study suggests that a guideline driven DHI CR program can augment secondary prevention strategies | | | | | | | | | | | | | | | | | |
| 24 | Orgen et al, 2018 | DI/UC | 660 (59%) | 69.6 | Sweden | Telemonitoring | | (36 months) | LDL, SBP, DBP | | IS, TIA | Cell Phone | | (NAILED) | Cognition, Persuasion | |  |
| **Note on Author's Study Summary**: Study intervention improved BP and LDL-C levels and increased the proportion of patients that reached the treatment target 36 months after discharge | | | | | | | | | | | | | | | | | |
| 25 | Zheng et al, 2019 | (Multicentre, Single-blinded Study), DI/UC | 822 (85.9%) | 56.4 | China | Telerehabilitation | | (6 months) | BMI, LDL, SBP, Medication adherence, Physical Activity, Smoking | | CHD | Cell Phone | | (CHAT) | Self- management, Persuasion, Motivation, Support | |  |
| **Note on Author's Study Summary**: Study affirms feasibility in the use of mobile phone SMS | | | | | | | | | | | | | | | | | |
| Qualitative Summary of Outcomes for Included Studies.  DI = Digital Intervention, UC = Usual Care, DHI = Digital Health Intervention, CR = Cardiac Rehabilitation, HbA1c = Haemoglobin A1c; ^a^ Diagnosis abbreviation available in appendices.  BMI = Body Mass Index, SBP = Systolic Blood Pressure, DBP = Diastolic Blood Pressure, TC = Total Cholesterol, HDL = High Density Lipoprotein, LDL = Low Density Lipoprotein, TG = Triglycerides. | | | | | | | | | | | | | | | | | |
